# Pupil Size Reflects Trial‐Level Variability in Imagery Vividness During Immersive Storytelling but Not (or Hardly) Individual Differences in Trait Imagery

**DOI:** 10.1111/psyp.70298

**Published:** 2026-04-10

**Authors:** Claire Vanbuckhave, Jakob Scherm Eikner, Bruno Laeng, Luca Onnis, Sebastiaan Mathôt

**Affiliations:** ^1^ School of Psychology University of Plymouth Plymouth UK; ^2^ Laboratoire de Psychologie et NeuroCognition CNRS Grenoble France; ^3^ Department of Linguistics and Scandinavian Studies University of Oslo Oslo Norway; ^4^ Department of Psychology University of Oslo Oslo Norway; ^5^ Department of Experimental Psychology University of Groningen Groningen the Netherlands

**Keywords:** narratives, pupillary light response, pupillometry, visual mental imagery, vividness

## Abstract

Previous research has shown that the eyes' pupils are larger when imagining dark as compared to bright objects or scenes. On the basis of this, it has been claimed that pupil size is a sensitive marker of mental imagery vividness. We investigated this claim in three experiments, conducted in two countries (Norway and The Netherlands; *N*_total_ = 115), in which participants read, listened, or freely imagined stories that evoked a sense of darkness or brightness. In addition, self‐reports of vividness were collected for each story to measure variations in imagery vividness during the experiment; and through questionnaires (VVIQ, SUIS), to measure differences in task‐unrelated imagery abilities at the individual level. We found that the effect of larger pupils for darkness‐evoking stories than brightness‐evoking stories was highly variable. Importantly, we found that this pupil‐size difference (dark–bright) was consistently largest for vividly imagined stories. Finally, we did not find any convincing relationship between this pupil‐size difference and individual differences in questionnaire‐based imagery. We conclude that the strength of pupil‐size changes in response to imagined darkness or brightness better reflects trial‐by‐trial fluctuations in imagery vividness within an individual than individual differences in imagery vividness as a personal trait.

## Impact Statement

Across three pupillometry experiments, this study shows that pupil size can track trial‐by‐trial fluctuations in mental imagery vividness during narrative processing of brightness‐related content. In contrast, pupillary responses show little to no association with questionnaire‐based imagery ability. By demonstrating that pupillary responses primarily reflect dynamic cognitive states, the findings refine and nuance the interpretation of imagery‐induced pupil size effects as a psychophysiological index of individual differences in mental imagery abilities.

## 1 Introduction

In recent years, there has been a growing interest in finding a reliable measure of visual mental imagery, defined as “representations and the accompanying experience of sensory information without a direct external stimulus” (Pearson et al. 2015, 590). In order to offer an objective window into such processes that are otherwise purely subjective, it is necessary to find a measurable response that operates without the need for an explicit report. Most approaches pursuing this objective build on the idea that visual imagery functions as a weak form of visual perception (Kosslyn et al. 2007; Pearson 2019) and are grounded in the well‐established parallels between visual perception and visual imagery (Ganis and Schendan 2011). A striking example of shared mechanisms between imagery and perception is that both seeing with the mind's eye and with the bodily eye affect the size of the pupil (Kay et al. 2022; Laeng and Sulutvedt 2014; Mathôt et al. 2017; Sulutvedt et al. 2018). Pupillometry has therefore recently emerged as a promising method to capture the sensory strength of imagined experiences at the physiological level.

Numerous studies show that pupil size varies consistently with the properties of an imagined object or scene. For example, the pupil is larger when imagining emotional scenes as compared to neutral scenes (Henderson et al. 2018). Pupil size is also affected by the size, distance (Sulutvedt et al. 2018), or brightness (Laeng and Sulutvedt 2014) of imagined objects. In a series of experiments, Laeng and Sulutvedt (2014) demonstrated that pupils became smaller when participants imagined increasingly brighter triangles compared to darker ones, after these had been previously seen. This effect was also observed when participants read or listened to single words (Mathôt et al. 2017; see also Mathôt et al. 2019; Xie and Zhang 2023) or imagined familiar scenes (Experiment 5 in Laeng and Sulutvedt 2014), typically associated with different levels of brightness (e.g., imagine a moonless night sky versus a bright sunny day). More recently, because imagining bright or dark triangles did not trigger such pupillary changes in individuals with aphantasia, it has been suggested that pupillary differences may serve as a physiological marker of visual mental imagery abilities (Kay et al. 2022).

Existing paradigms typically use minimalistic stimuli (e.g., triangles and single words) to maximize experimental control and may therefore not capture the richness of everyday imagery experiences. We hypothesized that this may explain why published effects tend to be small and variable; for example, although Mathôt et al. (2017) reported a reliable group‐level difference, around one third of participants showed an effect in the opposite direction, such that pupils were largest when reading brightness, as compared to darkness‐related words. Although it is possible that this variability reflects true individual differences, it seems more likely that it reflects measurement noise. Clearly, a marker of individual differences in mental imagery needs to be more reliable, and using naturalistic stimuli may be one way to achieve this. We therefore focused on short stories that conveyed a sense of brightness or darkness. We reasoned that narratives, by engaging richer and more immersive mental imagery than simple words (Mathôt et al. 2017) or geometric shapes (Laeng and Sulutvedt 2014), would enhance the magnitude and reliability of the imagery‐induced PLR. Three experiments were conducted independently and simultaneously by two separate research groups (in Norway and the Netherlands), who decided to combine their data post hoc in the present report.

In a first experiment, participants read narratives depicting bright or dark scenes (Experiment 1). In two further experiments, participants listened to short (Experiment 2) or longer (Experiment 3) audio stories, also depicting bright or dark scenes. Note that both these situations mimic common and natural exposure to narratives, either by reading text on a page or screen or by listening to audiobooks or radio programs. In Exp. 3, participants were also instructed to freely imagine scenes evoking a sense of brightness or darkness. Visual imagery abilities were assessed through vividness ratings for each story (as a measure of trial‐by‐trial variability) and standardized questionnaires after the experiment (as a measure of individual trait differences).

To foreshadow the results, overall, we found that darkness‐evoking stories induced larger pupils than brightness‐evoking stories, but only for audio stories presented in the third experiment. Importantly, we consistently found that this pupil‐size difference (dark–bright) was largest for vividly imagined stories. Finally, we did not find any relationship between this pupil‐size difference and individual differences in trait imagery.

## 2 Experiment 1

A previous study by Mathôt et al. (2017) demonstrated that reading single words could influence pupil size: words like *“bright”* caused pupils to constrict compared to *“dark”* words. The present study builds on this finding by examining the effect in a more ecologically valid context, where participants read entire narratives with a sense of brightness or darkness. As readers become immersed in the stories, they are expected to construct mental representations that mirror the light conditions described in the text, which should, in turn, elicit corresponding pupil responses.

### 2.1 Methods

#### 2.1.1 Participants

The target sample size for this Experiment was set to 50 participants, as recommended by Laeng and Mathôt (2024, 384) in order to achieve approximately 80% statistical power for the comparison of two within‐subject conditions in pupillometry studies. Fifty‐two naive individuals completed the experiment, recruited via posters advertising the experiment around the campus of the University of Oslo. Participants had to be aged between 18 and 35, speak Norwegian as a first language, and have normal or corrected‐to‐normal vision, free from any eye illnesses. Participants received a 150 NOK gift card upon completion.

After data preprocessing (see Preprocessing section below), the final sample comprised 50 individuals (age in years: *M* = 26.08, SD = 3.92). From these, 27 were identified as female (age in years: *M* = 25.89, SD = 4.03) and 23 as male (age in years: *M* = 26.30, SD = 3.85).

#### 2.1.2 Materials

Pupil diameter (right eye) was recorded using a Tobii spectrum eyetracker with a sampling rate set at 60 Hz. During the experiment, both the screen and the participant's head were shaded under a dark cloth, leaving only the computer screen (screen resolution: 1920 × 1080 pixels; refresh rate 60 Hz; model Eizo, EV24151) visible in the room (illumination constant at 179 lx). A chin rest stabilized the participant's head during reading at 55.5 cm from the screen. The experiment was programmed using the Tobii Pro Lab (version 1.232.52429).

#### 2.1.3 Stimuli

The stimuli consisted of four stories, divided into 16 paragraphs (4 neutral, 6 bright, 6 dark). Stories were generated with the assistance of UiO‐GPT, which provided the initial drafts that were then manually edited to prevent an excessive concentration of brightness‐related words. Each story depicted a protagonist moving through a series of fictional but realistic environments. All stories were presented in Norwegian to minimize effects associated with reading in a second language, such as potential reductions in mental imagery (Hayakawa and Keysar 2018) and increased cognitive effort (Borghini and Hazan 2018). Across paragraph types (neutral, bright, dark), the words did not differ on five lexical characteristics known to affect reading patterns: (1) lemma concreteness ratings (Brysbaert et al. 2014); (2) arousal ratings estimated from ChatGPT (Martínez et al. 2024); (3) lemma frequencies from the NoWaC corpus (Guevara 2010); (4) word length in characters; and (5) counts of nouns and verbs (see Appendix A: Supporting Information).

#### 2.1.4 Procedure

**FIGURE 1:**
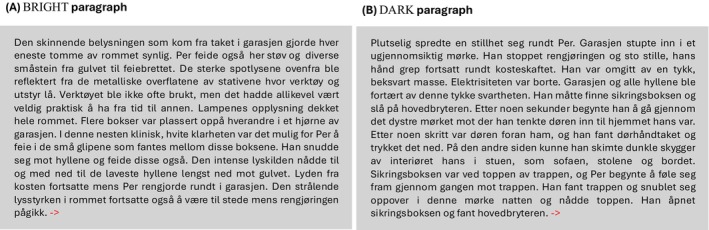
Example of experiment screens while reading a bright (A) or dark (B) paragraph, presented one after the other.

Each session began with a nine‐point calibration phase. After the calibration phase, a slide with instructions was presented, informing the participants that they were about to read four stories and that they should read as naturally as possible. An example of two experiment slides is provided in Figure 1.

Every short story started with a neutral paragraph (screen) for the participants to ‘settle’ into the story and read from the screen, followed by long paragraphs, covering about the whole screen, referring to bright and dark scenarios. In the latter, keywords spaced within the text referred to either the brightness or darkness of a scene (e.g., “driving on the road on a sunny day” or “driving through a dark tunnel”). All paragraphs consisted of dark text over a light gray background. A small red arrow at the end of each paragraph reminded participants to press the spacebar to continue to the next screen (self‐paced).

The presentation order was counterbalanced: half of the participants first read two stories in the neutral → bright → dark → bright paragraph order, followed by two stories in the neutral → dark → bright → dark paragraph order, whereas the other half did the opposite. After each story, a pause slide appeared, suggesting to the participants that they could have a rest.

Once the experiment was completed, participants responded to four yes/no questions assessing their memory for specific events and details from the previously presented stories (see Appendix B: Supporting Information for all four questions). Next, they reported how vividly or realistically (‘*levende*’) they could imagine each of the main characters in the stories on a Likert scale from 1 = ‘not at all’ to 7 = ‘a lot’. Participants also reported how much suspense or excitement (‘*spenning*’) they felt reading each story (from 1 = ‘not at all’ to 7 = ‘a lot’), in order to have a measure of both engagement and arousal elicited by the narratives (Kuijpers et al. 2021).

#### 2.1.5 Preprocessing

##### 2.1.5.1 Gaze Correction

Eye‐movement recordings are prone to drift, such that the recorded eye position becomes progressively less accurate as the trial progresses. In reading tasks, this can make it difficult to determine which word the participant is fixating on. Therefore, we removed drift from the gaze data (gaze correction) using the newly developed software *Fix8* (Al Madi et al. 2025) to acquire gaze corrected coordinates which were then applied to the data using R (version 4.3.3; R Core Team 2024). One participant was excluded from further analyses because their data could not be gaze corrected. The R datatable containing gaze‐corrected data was then exported as a .csv file and imported into Python (version 3.11.13) for the remaining preprocessing steps.

#### 2.1.6 Blink Reconstruction

Blink reconstruction was performed on the pupil‐size data during story reading using the advanced blink‐reconstruction algorithm implemented in Python DataMatrix (version 1.0.16). The function detects blinks on the basis of velocity thresholds applied to a lightly smoothed pupil trace (default is a 21‐ms Hanning window) and reconstructs the missing segments using cubic‐spline interpolation, or linear interpolation when insufficient boundary points are available (Mathôt and Vilotijević 2022).

Trials with more than 50% missing data (*n* = 6/612) after blink reconstruction, as well as trials with excessively low reading durations (*n* = 4/612 trials; ranging from 0.12 to 2.6 s) were excluded.^1^ One participant was also excluded for showing excessively high reading durations in all trials (*M* = 119.07 s, SD = 12.52 s, ranging from 101 to 144 s). Remaining missing values due to signal loss or unreconstructed blinks were linearly interpolated from the onset of the screen to the end of the reading phase, followed by a final smoothing step using a 51‐ms Hanning window to reduce residual jitter in the data. For each participant, trials with mean pupil sizes during story reading that were more than two standard deviations above or below the participant's mean (*n* = 13/612 trials) were also excluded. No participant had fewer than 50% valid trials remaining after these exclusion steps (final *N* = 50). Finally, pupil traces were baseline‐corrected by subtracting the median pupil size during the first 50 ms of each story from the entire pupil‐size time series.

### 2.2 Variables

We used two dependent variables for the analysis below. *Pupil‐size means* were calculated by averaging the baseline‐corrected pupil sizes over the full duration of paragraph reading for each trial. For each story and each participant separately, *pupil‐size mean differences* were obtained by subtracting the mean pupil size for the bright condition from the mean pupil size for the dark condition (i.e., a positive difference indicated that the pupil was greater during the reading of a story conveying a sense of darkness).

We used the following independent variables: Vividness and suspense ratings on the corresponding post‐experimental questionnaire items for each story, as well as by‐participant mean vividness, mean suspense, and mean accuracy ratings on the four memory questions.

### 2.3 Statistical Analyses

Mixed linear model analyses were conducted using the Python library *statsmodel* (version 0.14.1). All models were initially run with random by‐participant intercepts and slopes when appropriate. If a model failed to converge, we reverted to a simpler model with random by‐participant intercepts only. Assumptions checks were conducted for each model, using visual inspection (Q‐Q Plot) and Shapiro–Wilk's test (*scipy*, version 1.12.0) for normality of the residuals and White's Lagrange Multiplier test for homogeneity of variances (*statsmodel*, version 0.14.1). The effect of brightness level on the pupil measures was tested with pupil‐size means as the dependent variable and brightness level as a categorical factor (formula: *mean_pupil ~ brightness + (1 + brightness | participant))*.

The interaction between Vividness and Brightness was tested using two different approaches. The first approach was a ‘trial‐level’ approach, which consisted of adding the trial‐by‐trial vividness ratings as a linear predictor in interaction with the brightness level factor in the previous formula, for example, *mean_pupil ~ brightness* × *vividness + (1 + brightness | participant)*; this analysis tests whether the effect of imagined brightness on pupil size is mediated by systematic vividness differences between either participants or stories.

The second approach takes the pupil‐size differences between the bright and dark conditions as the dependent variable and the mean vividness rating scores across the bright and dark versions of each story as the independent variable (formula: *pupil_change ~ mean_vividness + (1 + mean_vividness | participant))*; this analysis tests whether the effect of imagined brightness on pupil size is mediated by differences between participants in vividness ratings, and is thus more directly related to individual differences. Missing values were excluded from each analysis.

Finally, to directly test the relationship between mean scores at the individual level, one‐sided Spearman's rank correlations with corresponding Bayes factors (Jeffrey's exact Bayes Factor; Ly et al. 2016) were calculated using the *pingouin* package (version 0.5.5) and coefficients interpreted following common guidelines (Akoglu 2018). Pupil‐size differences and self‐reported measures were averaged in order to obtain individual scores for each participant.

### 2.4 Results

#### 2.4.1 Descriptives

On average, a paragraph was read in 44.38 s (SD = 16.51; ranging from 17 to 180 s; 95th percentile = 75.54 s), and the mean accuracy per participant on the four memory questions was 77.00% (SD = 19.45%, chance = 50%). The mean vividness ratings score on the corresponding post‐experimental questions was *M* = 4.91 (SD = 0.96, range 2.0–7.0, IQR = [4.45, 5.5]) and *M* = 4.18 (SD = 1.02, range 1.25–6.0, IQR = [3.56, 5.0]) for the questions regarding Suspense.

#### 2.4.2 Pupil‐Size Means When Reading Fictional Narratives

##### 2.4.2.1 Overall, Pupil‐Size Means Do Not Differ Between the Bright and Dark Conditions at the Trial Level

**FIGURE 2:**
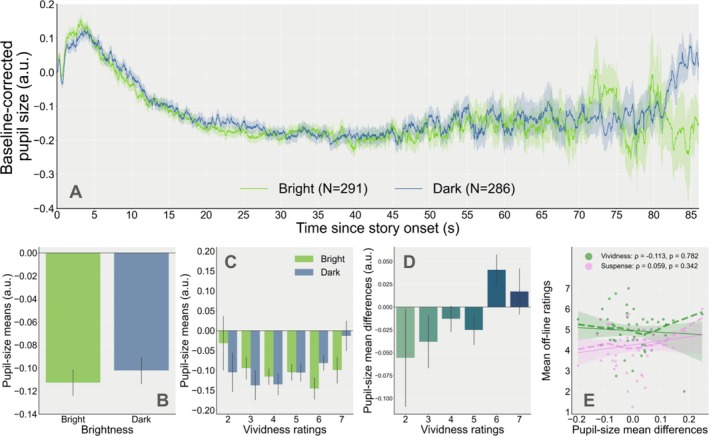
(A) Baseline‐corrected pupil‐size traces during the reading of the stories for each Brightness condition (except Neutral) as a function of time. The number of trials for each condition is indicated in parentheses in the legend. (B) Mean pupil size by brightness condition. (C) Pupil‐size means as a function of vividness ratings and brightness conditions. (D) Pupil‐size mean differences per story (mean pupil size during dark minus bright stories) as a function of vividness ratings. (E) Relationship between mean ratings (Vividness: Green; Suspense: Pink; averaged across stories for each participant) and pupil‐size mean differences. Each point of the same color represents scores for one participant (solid line: Fitted linear regression (robust) slopes; shaded area: 95% confidence intervals; dashed lines: Locally weighted linear regression slopes).

After visual inspection of the pupil traces (Figure 2A), it was unclear whether pupil size differed between Brightness conditions, although there was a weak tendency toward greater pupil sizes for DARK scenes as compared to BRIGHT scenes. Unsurprisingly, the main effect of the Brightness condition on pupil‐size means failed to reach significance at the trial level (M1: *β* = 0.008, SE = 0.014, 95% CI = [−0.018, 0.035], *z* = 0.605, *p* = 0.545, *n* = 50; Figure 2B).

##### 2.4.2.2 Vividness Ratings Modulate the Effect of Brightness Condition on Pupil‐Size Means at the Trial Level

When adding the interaction with off‐line vividness ratings of the stories in the model, the main effect of Brightness was still not significant (M2: *β* = −0.102, SE = 0.057, 95% CI = [−0.213, 0.009], z = −1.798, *p* = 0.072). However, a significant interaction revealed that pupil size during the BRIGHT trials became slightly smaller as compared to the DARK trials when Vividness increased (M2: *β* = 0.023, SE = 0.011, 95% CI = [0.001, 0.045], *z* = 2.010, *p* = 0.044, *n* = 50; Figure 2C). This second model better fitted the data compared to the simpler model (Log‐likelihood ratio test: *χ* ^2^(2) = 12.938, *p* = 0.0016, LLFM1 = 191.194, LLFM2 = 197.663). This effect could not be attributed to systematic differences in suspense, although including paragraph presentation order as a covariate significantly improved the model's goodness of fit (see Appendix A: Supporting Information Analyses). Although Figure 2D visually suggests that mean pupil‐size differences tended to be greater for stories rated as more vivid on average, this effect did not reach statistical significance (M3: *β* = 0.103, SE = 0.055, 95% CI = [−0.006, 0.212], *z* = 1.857, *p* = 0.063, *n* = 50).

##### 2.4.2.3 No Relationship at the Individual Level

At the individual level, mean Vividness scores, averaged across the four story pairs, revealed a moderate positive relationship with mean Suspense ratings (*ρ* = 0.423, 95% CI = [0.21, 1.00], *p* = 0.001, BF10 = 33.631, power = 0.931, *n* = 50). Looking at pupil‐size mean difference scores averaged across all story pairs, we observed that only 50.0% (25/50) of all participants had positive pupil‐size mean differences between the DARK and BRIGHT conditions at the individual level and found no significant correlations between mean pupil‐size differences and mean Vividness (*ρ* = −0.113, 95% CI = [−0.34, 1.00], *p* = 0.782, BF10 = 0.105, power = 0.008) or Suspense (*ρ* = 0.059, 95% CI = [−0.18, 1.00], *p* = 0.342, BF10 = 0.25, power = 0.108, *n* = 50; Figure 2E), with moderate evidence toward the null hypothesis that there is no correlation. Supplementary within‐story analyses revealed a weak positive correlation for Story 1, supported by anecdotal evidence (*ρ* = 0.304, 95% CI = [0.04, 1.00], *p* = 0.03, BF10 = 2.122, power = 0.604; see Appendix A: Supporting Information). Because mean accuracy was relatively low for yes/no questions, we also tested the relationship between mean pupil differences and mean accuracy on the four memory questions of the post‐experimental questionnaire. We found moderate evidence for a positive, albeit weak, correlation (*ρ* = 0.364, 95% CI = [0.14, 1.00], *p* = 0.005, BF10 = 9.284, power = 0.84, *n* = 50).

### 2.5 Discussion

In this experiment, participants read four short stories depicting either bright or dark environments, whereas their pupil size was continuously recorded. An effect of imagery‐induced semantic brightness was observed at the trial level, but only for trials that participants later rated as highly vivid. No reliable effect emerged at the story or participant levels. These findings suggest that vividness plays a modulatory role in the pupillary response to semantic brightness during narrative reading, although the effect was smaller and less robust than anticipated.

Despite applying gaze correction to the pupil data, the reading task inherently involves numerous motoric and visual factors such as saccades, fixations, and accommodation changes, which can interfere with the PLR (Mathôt and Vilotijević 2022). In addition, participants' reading speed and strategy could not be fully controlled (e.g., some may have skimmed, reread, or previewed text segments), and no fixation screen was presented between paragraphs, which may have influenced baseline pupil size. Additionally, the fact that some participants did poorly on the memory questions and the finding that greater pupil size differences were associated with higher mean accuracy scores could suggest that participants only superficially engaged with the narratives. This could mean they were not sufficiently immersed in the stories to experience the effects of perceptually grounded imagery.

A further limitation concerns the assessment of vividness. Ratings were collected only at the end of the experiment, which constitutes an offline measure. Such ratings are known to have reduced predictive validity compared to trial‐by‐trial vividness assessments (Runge et al. 2017), as they are more likely to rely on a retrospective summary evaluation of one's imagery experience during each story. This delayed rating could also explain the lower accuracy, as participants were more likely to confuse stories or have difficulties remembering specific details. Moreover, vividness was assessed with a single question per story and focused on how well participants imagined the main character, rather than the entire scene, which may have introduced interpretive ambiguity. Experiments 2 and 3 address some of these limitations.

## 3 Experiment 2

Although there has been a tendency to focus on reading (Brosch 2018), listening to audio‐verbal information or audio narratives can also elicit visual mental images (and sometimes even more strongly; Winzenz 1988). The presentation of audio stimuli has the main advantage of preventing eye movements (i.e., by instructing participants to keep their eyes on a fixation point at the center of the screen) and pupil‐size changes related to the effective brightness of the visual cues that occur during reading (e.g., if the words presented visually have different lengths). Drawing from the second finding in Mathôt et al. (2017) showing that pupil size was consistent with the semantic brightness of single words presented auditorily, we expected that this effect would be enhanced with the presentation of audio short stories, inducing bright or dark mental images.

### 3.1 Methods

#### 3.1.1 Participants

Participants were 1st year Psychology students recruited through the University of Groningen recruitment platform (SONA). The inclusion criteria were (1) to be at least 18 years old, (2) not to be under neurological or psychiatric treatment, (3) to have normal or corrected‐to‐normal vision and hearing, and (3) to have sufficient knowledge of the English or Dutch language to be able to follow written instructions, answer written questions, and understand the short stories they were asked to listen to and imagine.

Compared to Experiment 1, the target sample size for the second experiment was set to 30 participants, on the basis of the sample size reported in Mathôt et al. (2017; OSF Preregistration: https://osf.io/fge36). Because of over‐registration, 35 adult volunteers completed the second experiment. After preprocessing and trial exclusion, one participant had too few trials left in each condition to be included in the analyses (see Preprocessing section). The final sample size, therefore, consisted of 34 participants (age: *M* = 19.97 years old, SD = 1.57; experiment language version: English: *n* = 20, Dutch: *n* = 14). Of these, 22 participants identified themselves as female (age: *M* = 19.77 years old, SD = 1.38) and 12 as male (*M* = 20.33 years old, SD = 1.87). On average, participants rated the quality of their vision and hearing as ‘very good’ (vision: *M* = 1.65, SD = 0.60; hearing: *M* = 1.97, SD = 0.72).

### 3.2 Materials

Pupil size (right eye) was recorded as a time series during listening to the scenarios using an EyeLink 1000 Plus (SR Research) at a sampling rate of 1000 Hz. Testing took place in a dimly lit room of the lab (illumination: 6 lx). Participants placed their chin on a support placed at 25 cm from the computer screen (screen resolution: 1920 × 1080 pixels; refresh rate: 100 Hz), and 20 cm from the EyeLink camera lens. The experiment was programmed with OpenSesame (version 4.0).

### 3.3 Stimuli

The eight stories presented during the experiment were original stories written in English by the authors for the purpose of the study and carefully translated into Dutch. The English and Dutch audio versions of the stories were obtained by recording a native Dutch speaker who is bilingual in English (one of the authors) narrating the stories. All stories were narrated by the same speaker to ensure that the Dutch and English versions were as similar as possible.

Two types of stories were presented in pairs: DYNAMIC and NON‐DYNAMIC stories. The three NON‐DYNAMIC story‐pairs, labeled as *Birthday Party*, *Lord of the Rings* (LOTR), and *Neutral*, each consisted of two stories with opposite brightness levels but similar content (see Appendix B: Supporting Information); that is, there was both a bright and a dark version of the *Birthday Party* story. Within each NON‐DYNAMIC audio story, the brightness depicted in the scene was the same across time: the story either depicted a DARK scene or a BRIGHT scene. The two content‐matched DYNAMIC stories depicted a sense of brightness that varied across time. One audio story initially depicted a DARK scene, and toward the end, it depicted a BRIGHT scene (DARK‐TO‐BRIGHT). For the second DYNAMIC audio story, it depicted a scene that is initially BRIGHT and becomes DARK toward the end (BRIGHT‐TO‐DARK). The two brightness conditions were matched as closely as possible in terms of duration, content, and arousal between story‐pairs.

### 3.4 Procedure

**FIGURE 3:**
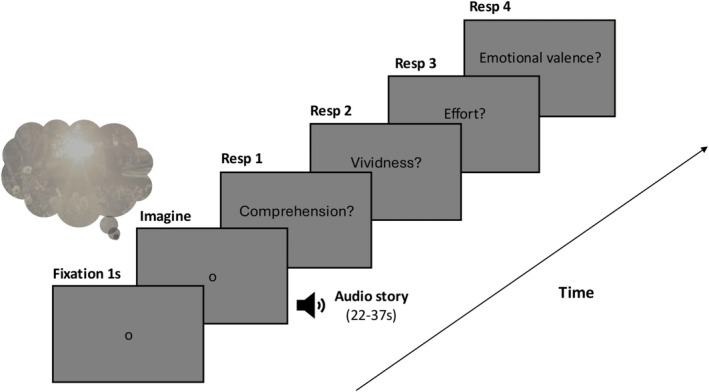
Illustration of the trial sequence in Experiment 2. Pupil size was recorded throughout the experiment. The stimuli consisted of four pairs of stories; the duration of the stories during the imagery phase was matched within pairs of stories.

A 5‐point calibration was performed at the beginning of the experiment. Each trial began with a one‐point drift correction, followed by the appearance of a dark gray (5.73 cd/m^2^) fixation dot in the center of the lighter gray screen (15.60 cd/m^2^) for 1 s (see Figure 3). The story was then presented auditorily while participants looked at the same fixation dot. On each trial, after the presentation of the story, participants answered four questions. We first presented a 3‐choice comprehension question to ensure that they were paying attention to the story and understood its meaning. This was followed by a question about the vividness of their imagery while imagining the stories: on a 5‐point scale ‘How clearly, vividly or realistically did you visualise the scenario in your head?’ (from 1 = ‘No image at all, you only “know” that you were thinking of the scene’ to 5 = ‘Perfectly clear and vivid as real seeing’). Pupil dilations can also occur in response to mental effort or arousal, for example, when completing a difficult task that requires attention or when being stimulated emotionally (Mathôt 2018). Hence, participants also answered an emotional valence question (‘What kind of emotions did you feel most while listening to the scenario?’; 7‐point scale from −3 = ‘very negative’ to +3 = ‘very positive’) and a mental effort question (‘How effortful was it to imagine this scenario?’; on a 5‐point scale from −2 = ‘very low effort’ to 2 = ‘very high effort’).

Afterward, participants completed a post‐experimental questionnaire which included the English and Dutch versions of the 16‐item Vividness of Visual Imagery Questionnaire (VVIQ; original: Marks 1973; Dutch translation: van der Aalst 2023) and the 12‐item Spontaneous Use of Imagery Scale (SUIS; original: Reisberg et al. 2003; Dutch translation: Nelis et al. 2014). The VVIQ requires participants to imagine various familiar scenes and to report how vividly the images that came up to their mind on a 5‐point scale (from 1 = ‘No image at all, you only “know” that you were thinking of the scene’ to 5 = ‘Perfectly clear and vivid as real seeing’). The SUIS focuses on the frequency and likelihood of mental imagery during everyday life, requiring participants to rate on a 5‐point scale how often a statement about their mental imagery use is appropriate for them (from 5 = ‘always completely appropriate’ to 1 = ‘never appropriate’; full questionnaires are available in Appendix B: Supporting Information). Finally, participants answered questions about their age, sex, quality of vision and hearing, and general understanding of the stories (related to language skills). The entire study was typically completed in 15–20 min.

### 3.5 Preprocessing

Data preprocessing was similar to Experiment 1. However, compared to Experiment 1, pupil size was then downsampled to 100 Hz right after blink reconstruction (original sampling frequency: 1000 Hz vs. 60 Hz in Experiment 1). Next, trials that contained an excessive number of blinks, reflected by a blink rate above two standard deviations from the mean blink rate for all trials (*n* = 12/280 trials, ranging from 35 to 56 blinks/min; *M* = 12.9 blinks/min, SD = 10.7 blinks/min) as well as trials with more than 50% of missing values (*n* = 4/280) were excluded. To ensure that the baseline period (0 to 50 ms after story onset) did not contain unreconstructed blinks and to minimize any artifacts it might contain, we linearly interpolated the missing pupil traces from the 200 ms before and after story onset (before the pupil light response latency, which is approximately 220 ms; Mathôt 2018), using the interpolation function of the same DataMatrix module. The pupil traces were then smoothed with a 51‐ms Hanning window to reduce the jitter in the data.

For each participant, trials with overall mean pupil sizes during the listening of the stories that were above or below two standard deviations from the mean were also excluded (*n* = 3/280 trials). One participant had less than 50% of trials remaining after these exclusion steps and was therefore also removed from the dataset (final *N* = 34). Finally, the pupil traces were baseline corrected by subtracting the median pupil size during the first 50 ms of each story from the pupil size time series.

#### 3.5.1 Variables

##### 3.5.1.1 Pupil‐Size Dependent Variables

The pupil‐size means and pupil‐size mean differences were computed following the same method described in Experiment 1. Additionally, we computed pupil‐size slopes by fitting a linear regression (*scipy* library, version 1.12.0) with pupil size as the dependent variable and time as the predictor to the pupil size time series. This was done for the BRIGHT‐TO‐DARK and DARK‐TO‐BRIGHT scenarios only, separately for each trial and after linear interpolation. Pupil‐size slope differences were obtained by computing individual differences between the DARK‐TO‐BRIGHT minus BRIGHT‐TO‐DARK scenarios.

##### 3.5.1.2 Reported Independent Variables

Mean scores of Vividness, Effort, Emotional Valence, and Emotional Intensity were calculated by averaging the corresponding trial‐by‐trial ratings across each story pair, for each story subtype and participant. Because trial‐by‐trial ratings did not specifically include a question about arousal, Emotional Intensity was defined as the absolute value of Emotional Valence. In addition to trial‐by‐trial ratings, mean questionnaire‐based scores (VVIQ and SUIS) were obtained by averaging responses across items for each participant.

### 3.6 Statistical Analyses

All analyses were again conducted in Python (*statsmodel* version 0.14.1) and initially run with random by‐participant intercepts and slopes when appropriate. Assumption checks were conducted following the methods described in Experiment 1. We once again ran the same three models as described in Experiment 1, once for pupil‐size mean variables and once for slope variables.

### 3.7 Results

#### 3.7.1 Descriptives

During the experiment, the mean accuracy per participant was 94.38% (SD = 8.35%, chance = 33.3%), with participants reporting having overall ‘Moderately clear and vivid’ mental images of the audio stories they were instructed to imagine (*M* = 3.35, SD = 0.86, range 2.38–4.5, IQR = [3.00, 3.61]). Both questionnaires showed good internal consistency (VVIQ: α = 0.853, 95% CI = [0.769, 0.916]; SUIS: α = 0.702, 95% CI = [0.529, 0.831]). The average score on the VVIQ was *M* = 3.53 (SD = 0.57, range 1.5–4.94, IQR = [3.32, 3.80]) and *M* = 3.34 on the SUIS (SD = 0.53, range 2.41–4.75, IQR = [2.92, 3.73], *n* = 34), which respectively corresponded to overall ‘Clear and reasonably vivid’ mental images and the use of visual imagery in various situations that was ‘Appropriate about half of the time’.

#### 3.7.2 Pupil‐Size Slopes for DYNAMIC Audio Stories

##### 3.7.2.1 Overall, Pupil‐Size Slopes Do Not Differ Between the Bright‐to‐Dark and Dark‐to‐Bright Conditions at the Trial Level

**FIGURE 4:**
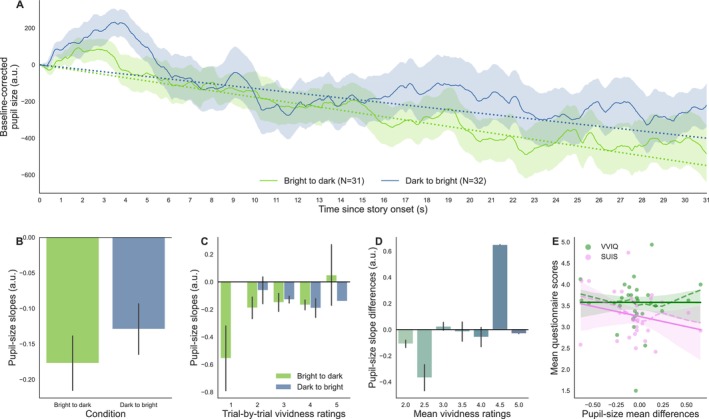
(A) Baseline‐corrected pupil‐size traces as a function of time during the imagining of the stories, split by condition (dark‐to‐bright and bright‐to‐dark conditions) for the Dynamic stories. Pupil traces are shown after linear interpolation. The number of trials for each condition is indicated in parentheses in the legend. The dotted lines represent each time‐sample multiplied by the mean slope values for each brightness condition, which were averaged across all participants. (B) Pupil‐size slopes averaged across participants by brightness condition. (C) Pupil‐size slopes as a function of vividness ratings and brightness condition. (D) Pupil‐size slope differences (value of the slopes during bright‐to‐dark minus dark‐to‐bright stories) as a function of vividness (trial‐by‐trial vividness ratings averaged across bright‐to‐dark and dark‐to‐bright stories). Variables were calculated individually for each participant. (E) Mean scores on the Vividness of Visual Imagery Questionnaire (VVIQ; green) and the Spontaneous Use of Imagery Scale (SUIS; pink) as a function of pupil‐size slope differences, in arbitrary units. Each point of the same color represents one participant's (individual) mean scores. The solid line and shaded area show the fitted linear regression (robust) lines and 95% confidence intervals; the dashed lines show locally weighted linear regression slopes.

For the DARK‐TO‐BRIGHT condition, we expected pupil size to be larger at the beginning of the story compared to the end, which would be reflected in our data by more negative pupil‐size slopes. For the BRIGHT‐TO‐DARK condition, we expected the opposite, with larger pupil size at the end of the story, that is, more positive pupil‐size slopes. Pupil‐size changes to baseline during both brightness conditions and their respective mean slope values (averaged across all participants for each brightness condition) are shown in Figure 4A. Contrary to our hypotheses, after visual inspection, pupil‐size slopes seemed slightly smaller for the BRIGHT‐TO‐DARK condition than the DARK‐TO‐BRIGHT condition for most participants. Statistically, this effect was not significant (M1: *β* = −0.054, SE = 0.048, 95% CI = [−0.148, 0.041], *z* = −1.110, *p* = 0.267, *n* = 30; Figure 4B).

##### 3.7.2.2 Vividness Ratings Modulate the Effect of Brightness Condition on Pupil‐Size Slopes at the Trial Level

When trial‐by‐trial ratings of vividness were added as a linear predictor to the model, we found a main effect of the Brightness condition suggesting that the pupil‐size slopes were significantly smaller for the BRIGHT‐TO‐DARK condition than for the DARK‐TO‐BRIGHT condition when Vividness was at its minimum (M2: *β* = −0.460, SE = 0.164, 95% CI = [−0.782, −0.138], *z* = −2.798, *p* = 0.005, *n* = 30). When a story was rated as more vivid, pupil‐size slopes then became greater for the BRIGHT‐TO‐DARK condition compared to the DARK‐TO‐BRIGHT condition, as indicated by the significant interaction between Brightness and Vividness (M2: *β* = 0.132, SE = 0.051, 95% CI = [0.031, 0.232], z = 2.564, *p* = 0.010, *n* = 30; Figure 4C). A likelihood ratio test confirmed that the second model, with Vividness as a linear predictor, better fitted our data than the first model (*χ* ^2^(2) = 7.136, *p* = 0.0282, LLFM1 = 9.633, LLFM2 = 13.201).

This interaction was also statistically significant when testing how individual ratings of vividness affected pupil‐size slope differences. Although only 30.0% (9/30) of participants had positive pupil‐size slope differences, slope differences became more positive for participants with higher vividness ratings (M3: *β* = 0.122, SE = 0.058, 95% CI = [0.008, 0.236], *z* = 2.099, *p* = 0.036). However, this effect seemed weaker than at the trial level and, after visual inspection, we cannot rule out the possibility that it may have been driven by a few individuals with especially strong effects (Figure 4D).

##### 3.7.2.3 No Relationship Between Pupil‐Size Slope Differences and Individual Scores on the Questionnaires at the Individual Level

The correlation between mean VVIQ and SUIS scores was not significant (*ρ* = 0.27, 95% CI = [−0.02, 1.00], *p* = 0.061, BF10 = 1.248, power = 0.468, *n* = 34). Similarly, we found no significant correlations between mean vividness ratings for these dynamic stories and the questionnaire scores (VVIQ: *ρ* = 0.204, 95% CI = [−0.11, 1.00], *p* = 0.14, BF10 = 0.677, power = 0.292; SUIS: *ρ* = 0.160, 95% CI = [−0.15, 1.00], *p* = 0.199, power = 0.214, BF10 = 0.506, *n* = 30). Once again, we found moderate evidence that participants with greater mean scores on the questionnaires did not necessarily show greater pupil‐size slope differences (VVIQ: *ρ* = −0.04, 95% CI = [−0.34, 1.00], *p* = 0.584, BF10 = 0.194, power = 0.032; SUIS: *ρ* = −0.257, 95% CI = [−0.52, 1.00], *p* = 0.915, BF10 = 0.103, power = 0.001, *n* = 30; Figure 4E).

Taken together, these results indicate that pupil‐size slopes, which reflect changes in pupil size over time, are increasingly consistent with the semantic brightness of an audio narrative depicting luminance changes when reported vividness increases. This effect appears to be more reliable at the trial level than the individual level and was limited to trial‐by‐trial ratings during the experiment.

#### 3.7.3 Pupil‐Size Means for NON‐DYNAMIC Audio Stories

##### 3.7.3.1 Overall, Pupil‐Size Means Do Not Differ Between the Bright and Dark Conditions at the Trial Level

**FIGURE 5:**
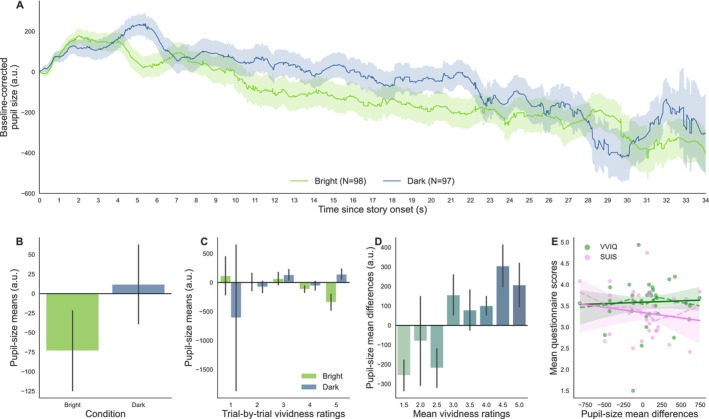
(A) Baseline‐corrected pupil‐size traces during the imagining of the stories (dark and bright conditions for all stories except Dynamic) as a function of time. The number of trials for each condition is indicated in parentheses in the legend. (B) Mean pupil size by brightness condition. (C) Pupil‐size means as a function of trial‐by‐trial vividness ratings and brightness condition. (D) Pupil‐size mean differences (mean pupil size during dark minus bright stories) as a function of mean vividness ratings (averaged across bright and dark stories for each story subtype and participant). (E) Relationship between mean scores on the questionnaires (VVIQ: Green; SUIS: Pink) and pupil‐size mean differences, in arbitrary units. Each point of the same color represents scores for one participant (solid line: Fitted linear regression (robust) slopes; shaded area: 95% confidence intervals; dashed lines: Locally weighted linear regression slopes).

The changes in pupil size relative to baseline while imagining the BRIGHT and DARK stories are shown in Figure 5A, all three NON‐DYNAMIC stories combined. Visually, it seemed that the tendency was in the expected direction, with overall greater pupil sizes for the DARK stories as compared to the BRIGHT stories. Yet, regardless of Vividness, we found no main effect of Brightness on mean pupil size at the trial level (M1: *β* = −60.673, SE = 56.901, 95% CI = [−172.197, 50.851], *z* = −1.066, *p* = 0.286, *n* = 34; Figure 5B).

##### 3.7.3.2 Vividness Ratings Modulate the Effect of Brightness Condition on Pupil‐Size Means at the Trial Level

**However**, when including trial‐by‐trial vividness ratings in the model, we found that for trials that were rated at the lowest level of Vividness, pupil‐size means during BRIGHT stories were in fact greater than during DARK stories (M2: *β* = 406.858, SE = 188.862, *z* = 2.154, *p* = 0.031, 95% CI = [36.695, 777.022], *n* = 34). Pupil size means during BRIGHT stories then became smaller as compared to DARK stories when the stories were rated as imagined more vividly (Figure 5C), as suggested by the significant interaction between reported Vividness and the effect of Brightness condition on the pupil‐size means (M2: *β* = −136.598, SE = 52.672, *z* = −2.593, *p* = 0.010, 95% CI = [−239.833, −33.363], *n* = 34). A model comparison with the likelihood ratio test confirmed that adding the vividness variable to the model better fitted the data (*χ* ^2^(2) = 7.488, *p* = 0.0237, LLFM1 = −1396.034, LLFM2 = −1392.29).

Similarly, looking at the effect of mean Vividness scores on pupil‐size difference scores computed for each story pair (LOTR, Happy Birthday, Neutral), pupil‐size differences increased i.e., became more positive, for story pairs that were rated as more vivid (M3: *β* = 150.878, SE = 71.138, *z* = 2.121, *p* = 0.034, 95% CI = [11.451, 290.305], *n* = 34; Figure 5D). After looking at other possible differences between conditions (e.g., blinks, emotional intensity, etc.), Supporting Information Analyses confirmed that it is unlikely that such non‐imagery related variables were responsible for the observed effects (see Appendix A: Supporting Information).

Individual pupil‐size difference scores averaged across the three story subtypes were positive for only 61.76% (21/34) of participants and did not significantly increase with greater mean vividness during the experiment (*ρ* = 0.206, 95% CI = [−0.09, 1.00], *p* = 0.121, BF10 = 0.717, power = 0.322, *n* = 34). In fact, supplementary analyses suggested that vividness effects may have been driven by one fictional narrative in particular, the Lord of the Rings story pair, for which we found extreme evidence toward the existence of a moderate positive relationship with mean pupil‐size differences at the individual level (*ρ* = 0.602, 95% CI = [0.37, 1.00], *p* = 1.4e‐04, BF10 = 248.941, power = 0.984, *n* = 32; see Appendix A: Supporting Information).

##### 3.7.3.3 No Relationship Between Pupil‐Size Mean Differences and Individual Scores on the Questionnaires at the Individual Level

Reporting higher levels of vividness on average during the whole experiment was moderately associated with higher mean VVIQ scores (*ρ* = 0.427, *p* = 0.006, 95% CI = [0.16, 1.00], power = 0.824, BF10 = 8.864, *n* = 34) but not higher mean SUIS scores (*ρ* = 0.277, *p* = 0.056, 95% CI = [−0.01, 1.00], power = 0.485, BF10 = 1.339, *n* = 34). However, participants with greater pupil‐size mean differences averaged across the three story pairs did not necessarily show greater mean scores on either of the questionnaires (VVIQ: *ρ* = 0.073, *p* = 0.34, 95% CI = [−0.22, 1.00], power = 0.11, BF10 = 0.303; SUIS: *ρ* = −0.174, *p* = 0.837, 95% CI = [−0.44, 1.00], power = 0.0041, BF10 = 0.115, *n* = 34; Figure 5E). Correlations between questionnaire‐based scores and pupil differences within each story subtype revealed a weak positive correlation with VVIQ scores for the Neutral story pair only, supported by anecdotal evidence (*ρ* = 0.314, 95% CI = [0.01, 1.], *p* = 0.046, BF10 = 1.683, power = 0.529; see Supporting Information Analyses for all comparisons).

### 3.8 Discussion

We tested the effect of visual mental imagery while listening to audio short stories depicting brightness or darkness on pupil size. Overall, contrary to our expectations, we found that pupil size overall was not significantly greater when the story depicted darkness compared to when it depicted brightness. Instead, we did find that this pattern depended on reported vividness, such that the more vivid a story was rated on a particular trial, the more pupil size aligned with the brightness level depicted in that story. Moreover, results suggest that pupil‐size responses to imagined brightness were more clearly modulated by trial‐by‐trial vividness ratings during the experiment rather than mean ratings or questionnaire scores. Correlation analyses revealed no significant association between offline measures of visual imagery, such as the SUIS and the VVIQ, and individual pupil‐change scores. Paired comparisons between brightness conditions revealed no systematic differences in terms of mental effort, arousal, blink rate, gaze position, or presentation order between bright and dark stories, hence reducing the possibility that the observed effects were driven by factors unrelated to visual mental imagery.

## 4 Experiment 3

In Experiment 2, we once again did not find a reliable overall main difference in pupil size between listening to brightness‐ or darkness‐related stories. Possibly, this is because the stories, which were very short, did not sufficiently evoke images of brightness or darkness. When reading a book or listening to a story, it might take some time to truly immerse ourselves in the imagination process and form vivid images in our mind's eye (Denis 1982). This third experiment was designed in order to investigate the effect of visual imagery on pupil size when listening to a longer, more immersive, fictional story, in the hope of finding a stronger overall effect on pupil size, and thus a higher sensitivity to detect individual differences. Additionally, and for the same reason, we explored the potential of ‘free’ trials, in which the content of the bright or dark scenes was up to each individual.

### 4.1 Methods

#### 4.1.1 Participants

A new group of 31 participants completed the third experiment, with the same target sample size rationale as for Experiment 2. All participants were recruited through the same platform as Experiment 2. The inclusion criteria were also identical, except that participants were only required to have sufficient knowledge of the English language but not the Dutch language. Because of a platform update that occurred during the completion of the post‐experimental questionnaires, the responses of one participant were lost and therefore absent from the statistics, including offline measures and demographics (Nquest = 30). From the 30 participants that submitted the questionnaire (age: *M* = 21.83 years old, SD = 2.99 years old), 19 identified as female (age: *M* = 21, SD = 2.87) and 11 as male (age: *M* = 23.27, SD = 2.76). On average, participants reported ‘very good’ vision and hearing abilities (vision: *M* = 1.70, SD = 0.60; hearing: *M* = 1.67, SD = 0.66).

#### 4.1.2 Materials

Pupil size recording, materials, and testing conditions (room, screen size and luminance, distance from screen, etc.) were exactly the same as Experiment 2.

#### 4.1.3 Stimuli

The audio stimuli presented during the first part of the experiment were the two parts of an original fiction written in English by the authors for the purpose of the study. The story consisted of a DARK and a BRIGHT part, each lasting about 2 minutes. To enhance the immersive qualities of the audio story, the narration was narrated by a native English speaker recruited through an online casting platform and was accompanied by a fairy musical background at a low intensity level. Taking into account participants' feedback during Experiment 2 (e.g., reporting the stories being ‘a bit fast‐paced’ or that they would appreciate a ‘more animated voice and tone’), the narrator followed a series of instructions in order to minimize such issues (stories and instructions were provided in Appendix B: Supporting Information). The story parts were matched as closely as possible in terms of duration, content, and intonation, and designed such that the presentation order of the DARK and BRIGHT parts was interchangeable, that is, the story made sense regardless of which part was presented first or second.

#### 4.1.4 Procedure

**FIGURE 6:**
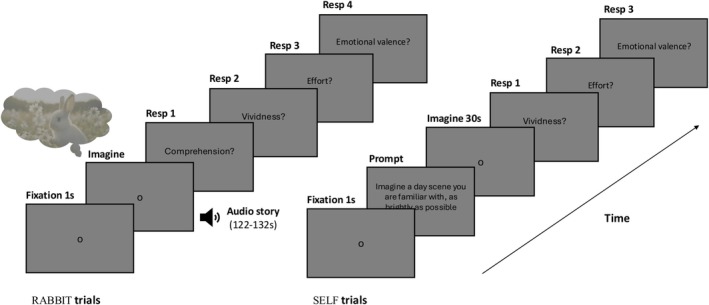
Illustration of the trial sequence for Experiment 3, with pupil size being recorded both during the 1‐s fixation phase and the imagery phase. The experiment consisted of two pairs of trials, and the duration of the imagery phase was matched within pairs of trials.

The procedure for the first two trials of the experiment was similar to the one described in Experiment 2, except that the presentation order of the stimuli was counterbalanced between participants rather than random (Figure 6). Half the participants began with listening to the part of the story that depicted a BRIGHT scene during the first trial and heard the second part depicting a DARK scene during the second trial. The other half of the participants listened to the story parts in the reverse order (a DARK scene followed by a BRIGHT scene).

After listening to the two‐part audio story, participants were given the opportunity to imagine a day or night scene of their choice, as long as they imagined it as BRIGHT or DARK as possible. Again, participants who started the experiment with the BRIGHT story‐part imagined a BRIGHT scene during their third trial (and a DARK scene during their fourth), whereas the other half were first instructed to imagine a DARK scene first, followed by a BRIGHT scene. Because we expected that participants would start picturing the scene in their minds while reading the instructions, the 1‐s fixation phase (baseline) preceded the instructions. Participants were instructed to press the space bar (or click) whenever they had a clear mental image of the scene to start the imagination phase, which initiated a 30‐s gray screen with the same fixation cross as before.

Similar to Experiment 2, four questions (Comprehension, Vividness, Mental Effort, and Emotional Valence) were presented after each trial, followed by the same post‐experimental questionnaire described in Experiment 2 after the experiment. Participants typically completed the entire procedure in 10–15 min.

#### 4.1.5 Preprocessing

The preprocessing steps differed from the ones described in Experiment 2 in two main points. The first point concerns the baseline period used to baseline‐correct the pupil‐size series, which was adapted to take into account the fact that participants started imagining the scenes while reading the instruction screen in the free trials. Hence, the baseline period consisted of the last 50 ms of the 1‐s fixation phase (vs. the first 50 ms of the imagery phase in Experiment 2).

The second point of divergence concerns the trial exclusion policy. Indeed, because the experiment included only four trials (two pairs with different instructions), we avoided trial exclusion as much as possible. Missing pupil‐size values could lead to failure in computing the baseline‐corrected signals or mean pupil sizes, and therefore increase the probability of a trial being excluded from the analyses. Hence, for each trial, missing values because of signal loss or unreconstructed blinks were linearly interpolated across using data from samples preceding and succeeding (i.e., from the onset of the presentation of the fixation cross to the end of the imagination phase). None of the trials contained more than 50% of missing values before interpolation, and the mean blink rate was very low (*M* = 8.733 blinks/min, SD = 8.787, 95th percentile = 27 blinks/min) such that trials with a blink rate above two standard deviations from the mean blink rate only ranged from 28 to 44 blinks/min (*n* = 7/124 trials). Therefore, these trials were not excluded from the analyses, but supplementary control analyses were conducted to check that the blink rate did not differ between Brightness conditions (see Appendix A: Supporting Information). Only outlier trials with overall mean baseline‐corrected pupil sizes above or below two standard deviations from the mean were excluded from the analyses (*n* = 5/124 trials). All participants had at least 50% of all trials remaining after preprocessing (final *N* = 31).

### 4.2 Variables

The computed variables were identical to the ones computed in Experiment 2 for the NON‐DYNAMIC stories (i.e., without pupil‐slope variables).

### 4.3 Statistical Analyses

Regarding the statistical analyses, the only deviation from Experiment 2 resided in the specification of the slopes and intercepts. As the presentation order of the stimuli had been counterbalanced between participants (i.e., each participant either started with the BRIGHT condition first or with the DARK condition first) and because pupil size is very likely to decrease as a function of time across an experimental session, especially with 2‐minute‐long trials, we also allowed different random intercepts and slopes per trial rank. Hence, the formula for the model was as: *Pupil ~ Brightness + (1 + Brightness + Rank | Participant)*. The interaction between Vividness and Brightness level was tested at both the trial and individual levels (trial‐level formula: Pupil ~ Brightness * Vividness + (1 + Brightness + Rank | Participant); individual‐level formula: *Pupil_Change ~ Mean_Vividness + (1 + Mean_Vividness + Order_Version | Participant)*).

### 4.4 Results

#### 4.4.1 Descriptives

During the experiment, the mean accuracy on the two comprehension questions related to the audio story was 96.67% (SD = 12.69%, chance = 33.3%), with participants reporting a very good general understanding of the narrative's content (*M* = 4.87, SD = 0.43) on the corresponding question during the completion of the post‐experimental questionnaires. The mean vividness ratings scores during the experiment suggested overall ‘Moderately clear and vivid’ mental images during the listening of the audio story (*M* = 3.23, SD = 0.85, range 1–4.5, IQR = [3.00, 3.50]) as well as during the free trials (*M* = 3.33, SD = 0.90, range 1–5, IQR = [3.00, 4.00]). The internal consistency was very good for both questionnaires (VVIQ: α = 0.940, 95% CI = [0.904, 0.967]; SUIS: α = 0.843, 95% CI = [0.746, 0.915], *n* = 30). On average, participants rated their imagery as ‘Moderately clear and vivid’ on the VVIQ (*M* = 3.35, SD = 0.81, range 1–4.63, IQR = [3.09, 3.77]) and reported that the statements provided in the SUIS were ‘Appropriate about half of the time’ (*M* = 3.20, SD = 0.73, range 1.33–4.42, IQR = [3.00, 3.67], *n* = 30).

#### 4.4.2 Pupil‐Size Means for the AUDIO Story

##### 4.4.2.1 Overall, Pupil‐Size Means Are Smaller in the Bright Condition as Compared to the Dark Condition at the Trial Level

**FIGURE 7:**
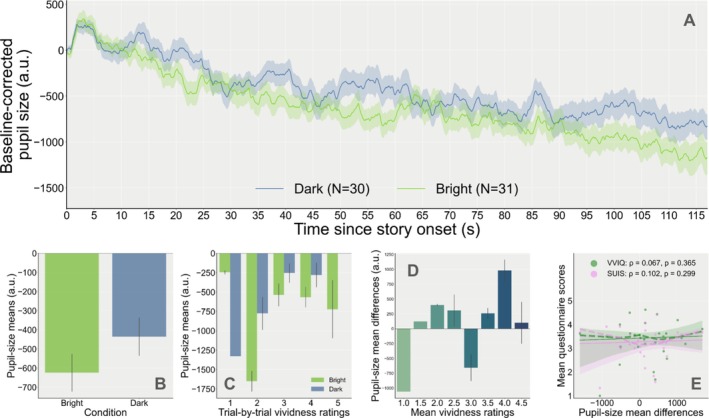
(A) Baseline‐corrected pupil‐size traces by brightness condition during the imagining of the story as a function of time. The number of trials for each condition is indicated in parentheses in the legend. (B) Mean pupil size for the bright and dark conditions. (C) Mean pupil size split by brightness condition and their corresponding self‐reported vividness level (trial‐by‐trial ratings). (D) Pupil‐size mean differences (mean pupil size during dark stories minus bright stories) as a function of mean vividness ratings (averaged across bright and dark stories), computed individually for each participant. (E) Relationship between individual pupil‐size mean difference scores and mean scores on the post‐experimental questionnaires (VVIQ: Green; SUIS: Pink; each dot of the same color represents one participant's scores; dashed lines: Locally weighted linear regression slopes; solid line: Slopes of the fitted linear regression (robust); shaded area: 95% confidence intervals).

Taking a look at how pupil size evolves as a function of time for each Brightness condition (Figure 7A), we can first notice a decrease in pupil size over time for both conditions; yet, overall, pupil size appeared greater when the story depicted a DARK scene as compared to a DARK scene. This effect seemed to arise around 10 s after the onset of the audio story and fluctuated across the trial, with more noticeable differences during the first 60 s of the story. Statistically, the main effect of the Brightness condition on pupil‐size means was indeed significant, with smaller mean pupil sizes when a story depicted a BRIGHT scene (M1: *β* = −417.380, SE = 132.414, *z* = −3.152, *p* = 0.002, 95% CI = [−676.906, −157.853], *n* = 31; Figure 7B).

##### 4.4.2.2 Vividness Ratings Modulate the Effect of Brightness Condition on Pupil‐Size Means at the Trial Level

When adding the interaction with vividness ratings in the model, however, the main effect of Brightness was no longer significant (M2: *β* = 423.056, SE = 462.306, *z* = 0.915, *p* = 0.360, 95% CI = [−483.047, 1329.159]), whereas the interaction was, such that when Vividness increased, pupil size during the BRIGHT trials became smaller as compared to the DARK trials (M2: *β* = −278.564, SE = 129.679, *z* = −2.148, *p* = 0.032, 95% CI = [−532.731, −24.398], *n* = 31; Figure 7C). A likelihood ratio test confirmed that the second model better fitted the data (*χ* ^2^(2) = 7.494, *p* = 0.0236, LLFM1 = −467.629, LLFM2 = −463.882).

At the individual level, only 66.67% (20/30) of all participants had positive pupil‐size mean differences between the DARK and BRIGHT conditions. Yet greater differences were observed for higher vividness ratings during the task (M3: *β* = 392.372, SE = 167.048, *z* = 2.349, *p* = 0.019, 95% CI = [64.964, 719.780], *n* = 30; Figure 7D). This effect could not be attributed to systematic differences in terms of blinks, emotional intensity, or mental effort (see Appendix A: Supporting Information).

##### 4.4.2.3 No Relationship Between Pupil‐Size Mean Differences and Individual Scores on the Questionnaires at the Individual Level

The mean VVIQ and SUIS scores were strongly correlated (*ρ* = 0.758, 95% CI = [0.59, 1.00], *p* = 6.2e‐07, BF10 = 3.286e+04, power = 1.0, *n* = 30). Mean vividness ratings during the listening of the story showed moderate positive correlations with the VVIQ (*ρ* = 0.503, 95% CI = [0.23, 1.00], *p* = 0.003, BF10 = 18.313, power = 0.89) and the SUIS (*ρ* = 0.616, 95% CI = [0.38, 1.00], *p* = 1.9e‐04, BF10 = 191.541, power = 0.981, *n* = 29). However, greater mean pupil‐size differences were not associated with increased mean scores on the VVIQ (*ρ* = 0.067, 95% CI = [−0.25, 1.00], *p* = 0.365, BF10 = 0.308, power = 0.098) or SUIS (*ρ* = 0.102, 95% CI = [−0.22, 1.00], *p* = 0.299, BF10 = 0.366, power = 0.133, *n* = 29; Figure 7E).

#### 4.4.3 Pupil‐Size Means for the FREE Trials

##### 4.4.3.1 Overall, Pupil‐Size Means Do Not Differ Between the Bright and Dark Conditions at the Trial Level

**FIGURE 8:**
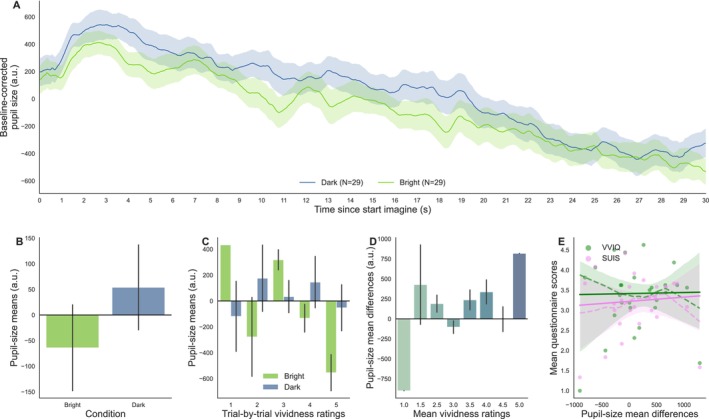
(A) Proportional pupil‐size changes relative to baseline as a function of time when participants were asked to imagine a bright or dark scene of their choice. The number of trials for each condition is indicated in parentheses in the legend. (B) Mean pupil size for the bright and dark conditions. (C) Mean pupil size split by brightness condition and their corresponding self‐reported vividness (trial‐by‐trial ratings). (D) Pupil‐size mean differences (mean pupil size during dark minus bright conditions) as a function of mean vividness ratings (averaged across bright and dark conditions), computed individually for each participant. (E) Relationship between individual pupil‐size mean difference scores and mean scores on the post‐experimental questionnaires (VVIQ: Green; SUIS: Pink; each dot of the same color represents one participant's scores; dashed lines: Locally weighted linear regression slopes; solid line: Slopes of the fitted linear regression (robust); shaded area: 95% confidence intervals).

As can be seen in Figure 8A, pupil size visually appeared greater when participants imagined a scene depicting darkness than when imagining a scene depicting brightness. On the screen prior to the start of the imagining phase, participants were instructed to first visualize the scene they want to imagine, and then: ‘once you have a clear picture in your mind, click to start imagining’. For this reason, small differences between Brightness conditions can be visually spotted from the onset of the imagination trial. The effect fluctuated throughout the trial, with short intervals that seemed to yield greater differences and appeared nearly non‐existent near the end of the 30‐s period. Statistically, regardless of Vividness, the effect of Brightness on mean pupil size averaged across the whole trial was not statistically significant at the trial level (M1: *β* = −143.357, SE = 99.113, z = −1.446, *p* = 0.148, 95% CI = [−337.615, 50.901], *n* = 30; Figure 8B).

##### 4.4.3.2 Vividness Ratings Modulate the Effect of Brightness Condition on Pupil‐Size Means at the Trial Level

Yet, after accounting for trial‐by‐trial Vividness into the model, compared to the DARK condition, we found that mean pupil size was greater when imagining a BRIGHT day scene at the lowest level of Vividness (M2: *β* = 664.510, SE = 303.649, *z* = 2.188, *p* = 0.029, 95% CI = [69.369, 1259.651]), and became smaller when Vividness increased (M2: *β* = −230.069, SE = 88.121, *z* = −2.611, *p* = 0.009, 95% CI = [−402.782, −57.355], *n* = 30; Figure 8C). The model including Vividness as a linear predictor in interaction with Brightness shows a significantly better fit to the data than the previous one (*χ* ^2^(2) = 6.786, *p* = 0.0336, LLFM1 = −434.051, LLFM2 = −430.658).

At the individual level, although only 60.71% (17/28) of all participants showed a positive pupil‐size difference score between Brightness conditions, significantly greater differences could also be observed for participants reporting more vivid or reality‐like mental images of the story (M3: *β* = 235.461, SE = 107.376, *z* = 2.193, *p* = 0.028, 95% CI = [25.009, 445.914], *n* = 28; Figure 8D). This effect could not be better explained by systematic differences in blink rate, emotional intensity, or mental effort (see Appendix A: Supporting Information).

##### 4.4.3.3 No Relationship Between Pupil‐Size Mean Differences and Individual Scores on the Questionnaires at the Individual Level

Participant's mean trial‐by‐trial vividness ratings across the experiment correlated moderately with their mean scores on the VVIQ (*ρ* = 0.625, 95% CI = [0.38, 1.00], *p* = 1.9e‐04, BF10 = 188.598, power = 0.981) but only weakly for the SUIS (*ρ* = 0.335, 95% CI = [0.02, 1.00], *p* = 0.041, BF10 = 1.896, power = 0.551, *n* = 28). However, pupil‐size difference scores did not directly correlate with either of the two questionnaires (VVIQ: *ρ* = 0.011, 95% CI = [−0.31, 1.00], *p* = 0.477, BF10 = 0.245, power = 0.056; SUIS: *ρ* = 0.11, 95% CI = [−0.22, 1.00], *p* = 0.289, BF10 = 0.384, power = 0.14, *n* = 28; Figure 8E).

### 4.5 Discussion

Pupil size was measured while participants listened and imagined a 2‐minute‐long brightness‐related audio fiction, before being asked to freely imagine a familiar scene conveying brightness or darkness. As expected, presenting more immersive, longer story parts induced greater differences between the bright and dark conditions, as suggested by a significant main effect of brightness condition in the first model. However, echoing the findings from the previous experiment, including vividness as a factor in the model better predicted pupil‐size differences, with greater differences when participants reported more vivid imagery. This was revealed both at the trial and individual levels, as well as when accounting for differences between brightness conditions in terms of blink rate or measures of mental effort and emotional intensity. Yet, we once again did not find any association with questionnaire‐based measurements.

## 5 General Discussion

Previous studies have shown that the pupil reacts to imagined light, such that the size of the pupil is smaller when having mental images of brightness‐related words (Mathôt et al. 2017), familiar scenes, or simple shapes (Laeng and Sulutvedt 2014; Kay et al. 2022) as compared to matching darkness‐related stimuli. However, the effect of imagined brightness or darkness on pupil size tends to be small and variable, and therefore not suitable as a measure of individual differences in imagery vividness. The main purpose of this research was to develop a more reliable way to elicit pupil‐size changes in response to mental imagery. To this purpose, we measured pupil size while participants read or listened to fictional stories depicting brightness or darkness, or while participants freely imagined a bright or dark familiar scene.

Contrary to our expectations, the effect of overall larger pupils for darkness‐ than brightness‐related stories remained small and variable in the current study (i.e., 50%–66.67% of all participants had a positive difference between dark and bright conditions) and was only statistically significant in Experiment 3. However, our results also suggest that a small—but statistically significant—part of the variability observed in pupil‐size differences between brightness conditions could be explained by trial‐to‐trial differences in how vividly a given participant visualized the content of the bright and dark narratives. Indeed, all results taken together, the most consistent finding was that, in all three experiments, the magnitude and direction of the imagery‐induced pupillary effect were modulated, albeit modestly, by ratings of vividness at the trial level. Although this confirms a relationship between trial‐level fluctuations in mental imagery vividness and the strength of corresponding bodily responses, such as pupil‐size changes (Kay et al. 2022; Wicken et al. 2021), several limitations should be discussed. First, despite our attempts to create ecologically valid and immersive stimuli, the modulation of imagery‐induced pupillary effects by vividness remained weaker than expected. Second, this relationship did not systematically extend to individual differences in mental imagery vividness. Specifically, participants who rated themselves as vivid imagers on post‐experimental questionnaires did not show overall stronger pupillary responses to darkness‐ or brightness‐related stories. These results suggest that pupil‐size changes were better predicted by how vividly specific items or pairs of items were imagined during the experiment than by one's general tendency to have vivid imagery (VVIQ) or to use visual imagery in daily life (SUIS).

Now perhaps this strong, hypothetical relationship between vividness and the pupil size that persists at both the trial and individual levels *does* exist, but we failed to capture it because of the presence of confounding variables that we did not account for. Indeed, keeping the experiments short and immersive while controlling for *every* possible variable in the present study was not feasible. Many factors can interfere with one's ability to imagine vivid images, such as restricted eye movements to the center of the screen (Ladyka‐Wojcik et al. 2022; but see: van den Hout et al. 2001) or the ability to maintain sustained attention and engagement in the task throughout a story (Rodero 2012). Although we have assessed the most common confounding variables in pupillometry experiments (e.g., mental effort, emotional intensity, blinks, properties of printed words), we did not use advanced cognitive screening, and it is also possible that other factors have attenuated the observed effects and prevented us from achieving the desired level of sensitivity. Other physiological factors may have directly mediated the observed effects, such as individual differences in sensory sensitivity to light (Dance et al. 2021), in ocular anatomical features (Sharma et al. 2016) or in spontaneous pupil‐size fluctuations (Ruuskanen and Mathot 2026).

Importantly, we did not present a visual illustration of the stories or scenes prior to the imagination phase, which means that there was no ‘normative’ or ‘reference’ visualization and that each participant could have conjured up very different visual images. In line with studies showing an association between vividness and the richness of the autobiographical memory (Milton et al. 2021; Zeman et al. 2020), differences in the availability of a relatable long‐term sensory trace to form the mental images or in memorability could therefore be a source of both between‐ and within‐individual variability (Cohen and Parra 2016; D'Angiulli et al. 2013). This aligns with our finding that the Lord of the Rings narratives showed the stronger vividness‐PLR association effects, as participants could have relied on shared memories of the movies to conjure up the associated mental images. Additionally, although the choice of having longer imagination phases was intended to induce stronger effects, the extent to which averaging pupil size over lengthy time windows still reflects pupillary light responses and not something else is questionable. That is, further experimentations are needed to help us better interpret individual variability in how pupillary light responses are induced by visual mental imagery.

Another explanation for why pupil‐size differences were hardly modulated by individual differences in imagery vividness is that perhaps such effects *do* exist, but are variable and therefore difficult to capture. This should not be surprising given the increasing number of studies failing to find a reliable relationship between individual‐level imagery vividness and more objective measurements (Azañón et al. 2025; Bouyer et al. 2025; Gardner et al. 2026; Vanbuckhave et al. 2026). At an alpha level of 5%, in the current study, we were only able to detect correlations above 0.30–0.45 with 80% statistical power given the sample sizes in each experiment. Studies with similar *N* to Experiment 1 have also failed to capture individual differences in reported vividness through variations in pupil size (Gardner et al. 2026; Kay et al. 2022), it is, therefore, theoretically possible that imagery‐induced PLR effects are inherently so small that hundreds of participants would be needed to reliably detect them across various experimental conditions (Laeng and Mathôt 2024). However, would that be enough? As a reviewer commented, beyond sample size, a lack of representativeness could also prevent us from detecting individual‐level variations. Indeed, our sample mostly comprised participants with typical imagery abilities and lacked individuals at the low and high ends of the imagery spectrum (Zeman 2024). In this case, it would call into question how sensitive PLR effects are to variations in experienced vividness. Perhaps the measurement resolution is such that it mostly captures low and high variations in vividness, such as the between‐group differences reported by Kay et al. (2022).

Relatedly, the validity of the vividness scales themselves could be questioned. Are there truly measurable differences in the vividness of experienced imagery between two individuals who report an overall score of 3 and 3.5 on a Likert scale? We cannot rule out the possibility that, within a group of individuals who report relatively similar imagery vividness, variations in reported vividness of visual imagery simply reflect differences in how participants think about themselves. In other words, even if the amplitude of the pupillary response to imagined brightness sensibly reflects one's ‘true’ experienced vividness of visual imagery, differences in how individuals consciously evaluate their own imagery abilities (Laeng et al. 2012) and how they use the provided scale to report them could weaken the relationship between the two variables. This is consistent with a recent unsuccessful attempt to replicate the results of Kay et al. (2022), which indicated that minor changes to the experimental procedure, such as providing additional verbal instructions on how to use Likert scales, could possibly influence the outcomes (Gardner et al. 2026). Although we did not provide any more specific instructions than those displayed on the screen, the fact that participants had not been asked about how vividly or clearly they visualized brightness or darkness features specifically, but rather how vividly they imagined the whole scene, may have introduced supplementary noise. Further investigations are required into the extent to which both the predictive power of subjective reports and the sensitivity of pupillometry protocols could be improved. This includes, for example, introducing a training session prior to the experiment to improve participants' metacognitive abilities regarding the vividness of their imagery (Rademaker and Pearson 2012), incorporating a non‐committal response option to reduce potential confabulation regarding their imagery properties (Bigelow et al. 2023) or other specific adjustments such as presenting a larger pool of stories, adjusting the vividness question to be more specific to brightness properties or using a more graded vividness scale.

To conclude, pupil‐size differences in response to imagined darkness or brightness can capture differences in reported imagery vividness; however, this relationship seems to hold only for fluctuations in imagery vividness within an individual; pupil‐size changes do not seem to be a sensitive marker of individual differences in imagery vividness as a personal trait.

## Author Contributions

**Claire Vanbuckhave:** conceptualization, methodology, software, visualization, investigation, formal analysis, writing – original draft writing. **Jakob Scherm Eikner:** conceptualization, methodology, software, investigation, data curation, writing – reviewing and editing. **Bruno Laeng:** supervision, resources, writing – reviewing and editing. **Luca Onnis:** supervision, writing – reviewing and editing. **Sebastiaan Mathôt:** software, validation, supervision, project administration, resources, writing – reviewing and editing.

## Funding

This work was partially (Experiments 2 & 3) supported by the ANR project ANR‐15‐IDEX‐02 and the MSH‐Alpes‐SCREEN platform of Grenoble Alpes University. Experiment 1 was supported by an internal grant from the EyeHub group, at the University of Oslo.

## Ethics Statement

All participants gave their informed written consent before participating in the study, which was in accordance with the APA Ethics Code (https://www.apa.org/ethics/). The data collection procedure respected the General Data Protection Regulation (GDPR), and the protocol had been approved by an ethical review board (ERB) prior to conducting the research.

## Conflicts of Interest

The authors declare no conflicts of interest.

## Supporting information


**Figure S1:** Distribution of reading durations across all trials, before outlier exclusion.
**Figure S2:** Mean scores per participant on the Vividness (left plot) and Suspense (right plot) post‐experimental questions.
**Figure S3:** Mean Vividness scores per participant, as obtained on the trial‐by‐trial ratings during the experiment (upper plot), or through post‐experimental questionnaires (VVIQ: lower left plot; SUIS: lower right plot).
**Figure S4:** Mean gaze position per brightness condition.
**Figure S5:** Mean Vividness scores per participant, as obtained on the trial‐by‐trial ratings during the experiment (upper plot), or through post‐experimental questionnaires (VVIQ: lower left plot; SUIS: lower right plot).

**Data S2:** Supporting Information.

## Data Availability

The data that support the findings of this study are openly available in GitHub at https://github.com/cvanbuckhave/pupil_stories_imagery.

## Endnotes

^1^
Excessively low reading times corresponded to durations below two standard deviations from the mean duration, that is, when a whole paragraph was read in less than 3 s (see Figure S1 in Appendix A: Supporting Information analyses for a visual representation of the reading time distribution).
