# Supplementary material for: Pupil Size Reflects Trial‐Level Variability in Imagery Vividness During Immersive Storytelling but Not (or Hardly) Individual Differences in Trait Imagery
Source: Psychophysiology. 2026 Apr 10;63(4):e70298. doi: 10.1111/psyp.70298 (PMC13067808; doi:10.1111/psyp.70298)
Supplement: Supplementary file 2 — Data S2: Supporting Information. [file PSYP-63-e70298-s002.pdf]

Appendix B: Supplementary materials

All materials (questionnaires, experiment files, stimuli, etc.), anonymised data (pupillometry and questionnaire data) and analysis scripts have been made publicly available at https://github.com/cvanbuckhave/pupil_stories_imagery.

Experiment 1

Instructions (English translation)

Translation of the instruction slide:

In this experiment, you will read 4 short stories about everyday situations. Your task is to read the texts calmly and at your own pace. You will have the opportunity to take a break between each story.

When you have finished reading the text on a page and reached this symbol →, you press the spacebar to proceed to the next page/slide.

After you have read all the stories, we will ask a few questions related to the content of the texts you have read.

When you are ready to begin you can press the spacebar on the keyboard.

Translation of break pages/slides:

That was the first/second/third story. You can continue reading another one when you are ready

Press the spacebar to continue.

Stimuli (original)

All narratives start with a neutral paragraph.

Narrative 1 & 2 has the narrative structure: bright – dark – bright.
Narrative 3 & 4 has the narrative structure: dark – bright – dark.

Target words are marked in **bold.**

Fortelling 1

Timestamp 1: start measuring pupil diameter

Nøytralt avsnitt. 147 ord, 0 målord

Ole våknet opp for bilturen som han hadde planlagt i flere dager. Han gikk ut av sengen og ned trappene i huset sitt, med hendene foldet sammen foran seg. Nede i stuen ved kjøkkenet forberedte han seg for turen ved å følge sine vanlige rutiner. Han så gjennom pakkelisten sin igjen og forsikret seg om at alle essensielle gjenstander var inkludert. Han markerte hver gjenstand som var skrevet på listen med et kryss. Ifølge listen skulle alt være pakket og lastet i bilen. Ole gikk til badet sitt, pusset tenner og fant toalettmappen sin hvor han kunne pakke sine siste gjenstander. Han pleide alltid å glemme noe, men ikke denne gangen. Med alt pakket og alle forberedelser fullført låste Ole hjemmet sitt. Han gikk til garasjen og åpnet den elektriske porten med en fjernkontroll. Han åpnet fordøren til bilen sin, satte seg inn og skrudde på motoren.

Timestamp 2: continue measuring pupil diameter

Lyst avsnitt. 151 ord: 6 sammensatte måleord, 10 diskrete måleord

Det var en **strålende sommerdag** da Ole kjørte på motorveien i bilen sin. Alt var klart for en tur som skulle vare i flere dager. **Solstrålene skinte** på bilpanseret mens han kjørte. Det nærmet seg flere svinger i veien foran ham og Ole bestemte seg av den grunn for å skifte gir med sin høyre hånd. Asfalten han kjørte på **skimret** i varmen foran ham. Ole kunne nå se fjerne fjell i horisonten. Det **blendende lyset** gjorde kjøringen gjennom svingene vanskeligere, men Ole var stødig bak rattet og kjørte kontrollert. Han hadde sin venstre hånd på rattet og hvilte ryggen sin mot bilsetet. Landskapet rundt ham nærmest **glinset** mens Ole fortsatte kjøringen og kom enda nærmere fjellene han så foran seg. Store steiner som lå oppover fjellene begynte å bli synlige mens Ole åpnet vinduet sitt for å slippe luft inn i bilen. Dette var absolutt en varm og **solfylt dag**.

Tidsstempel 3: fortsett måling av pupillediameter

Mørkt avsnitt. 148 ord, 6 sammensatte målord, 10 diskrete målord

Turen fortsatte og motorveien førte Ole videre mot et stort fjell og en **mørk tunnel** gjennom det. Ole plasserte sin høyre hånd på rattet og brukte sin venstre hånd for å lukke vinduet. Temperaturen sank da han nærmet seg den **sorte** inngangen. Veien gikk som en rett strek rett framover. Da Ole kjørte gjennom inngangen, begynte **svarte skygger** å dukke opp. Han passerte under to store vifter som lagde en dyp lyd som forsvant da han passerte dem. Ole kjørte gjennom en ugjennomtrengelig **bekmørk natt**. Han kjente gasspedalen under høyre fot og trykket den litt ned. Dette **dunkle skyggeområdet** var som en ny verden for ham. Ole trykket clutchen helt ned for å gjøre seg klar til å skifte gir med høyre hånd. Veggene rundt ham var dekket av et **kullsvart** lag med gammel eksos. Motoren ga fra seg en høyere tone mens veien begynte å gå oppover.

Tidsstempel 4: fortsett måling av pupillediameter

Lyst avsnitt. 152 ord, 6 sammensatte målord, 10 diskrete målord

Skilt langs veien indikerte at utgangen var nær, og Ole kunne se det **klare lyset** nærme seg i enden. Veien fortsatte enda i en rett linje framover, slik den hadde gjort en stund. Den **strålende, hvite** utgangen ble større ettersom Ole nærmet seg den. Han skiftet gir og akselererte, nålen på speedometeret steg sakte. I det øyeblikket Ole kjørte gjennom utgangen, ble han overveldet av **blendende dagslys** på den andre siden. Først var det vanskelig å se, men etter hvert begynte konturene i omgivelsene å dukke opp igjen. Det sterke **sollyset** la seg over hele landskapet og også inn i bilen til Ole. Han begynte å se trær og store steiner på fjellene rundt ham. Toppene på de fjerne fjellene var badet i en tydelig **gyllen glød**. Trærne var høye med greiner på toppen, og steinene var store og runde. Ole hadde fortsatt flere timer igjen å kjøre i disse **glitrende** omgivelsene.

Tidsstempel 5: stopp måling av pupillediameter

Fortelling 2

Nøytralt avsnitt. 150 ord, 0 målord.

Per dro kosten over teppet. Huset trengte å bli rengjort. Han fortsatte å feie gulvet mens han beveget seg fra stuen til gangen. Han feide smuler og dyrehår opp fra gulvet til et feiebrett. Lyden av kosten mot teppet ga fra seg en rytmisk lyd. Han flyttet seg til kjøkkenet, hvor han bevegde seg rundt spisebordet og stolene. Noen magasiner lå på den ene enden av bordet, og alle stolene hadde puter på seg som han nettopp hadde kjøpt i butikken. Per ble ferdig i dette rommet og bevegde seg mot garasjen med kosten fortsatt i hendene sine. Han passerte en stabel uåpnet post som lå på kjøkkenbenken. Inni garasjen var det rotete. Hyller, bokser og verktøy var rundt omkring. En stor verktøykasse med en hammer, noen skruer og mange forskjellige andre ting, sto åpen på gulvet. Han stoppet et sekund og så bort på de støvete hyllene og gulvet.

#2 tidsstempel: fortsett måling av pupillediameter

Lyst avsnitt. 150 ord, 6 sammensatte målord, 10 målord.

Den **skinnende belysningen** som kom fra taket i garasjen gjorde hver eneste tomme av rommet synlig. Per feide også her støv og diverse småstein fra gulvet til feiebrettet. De sterke **spotlysene** ovenfra ble reflektert fra de metalliske overflatene av stativene hvor verktøy og utstyr lå. Verktøyet ble ikke ofte brukt, men det hadde allikevel vært veldig praktisk å ha fra tid til annen. **Lampenes opplysning** dekket hele rommet. Flere bokser var plassert oppå hverandre i et hjørne av garasjen. I denne nesten klinisk, **hvite klarheten** var det mulig for Per å feie i de små glipene som fantes mellom disse boksene. Han snudde seg mot hyllene og feide disse også. Den intense **lyskilden** nådde til og med ned til de laveste hyllene lengst ned mot gulvet. Lyden fra kosten fortsatte mens Per rengjorde rundt i garasjen. Den **strålende lysstyrken** i rommet fortsatte også å være til stede mens rengjøringen pågikk.

#3 tidsstempel: fortsett måling av pupillediameter

Mørkt avsnitt. 147 ord, 6 sammensatte målord, 10 diskrete målord.

Plutselig spredte en stillhet seg rundt Per. Garasjen stupte inn i et **ugjennomsiktig mørke**. Han stoppet rengjøringen og sto stille, hans hånd grep fortsatt rundt kosteskaftet. Han var omgitt av en tykk, **beksvart** masse. Elektrisiteten var borte. Garasjen og alle hyllene ble fortært av denne tykke **svartheten**. Han måtte finne sikringsboksen og slå på hovedbryteren. Etter noen sekunder begynte han å gå gjennom det **dystre mørket** mot der han tenkte døren inn til hjemmet hans var. Etter noen skritt var døren foran ham, og han fant dørhåndtaket og trykket det ned. På den andre siden kunne han skimte **dunkle skygger** av interiøret hans i stuen, som sofaen, stolene og bordet. Sikringsboksen var ved toppen av trappen, og Per begynte å føle seg fram gjennom gangen mot trappen. Han fant trappen og snublet seg oppover i denne **mørke natten** og nådde toppen. Han åpnet sikringsboksen og fant hovedbryteren.

#4 tidsstempel: fortsett måling av pupillediameter

Lyst avsnitt. 151 ord, 6 sammensatte målord, 10 diskrete målord.

Fingrene hans vippet opp hovedbryteren, og umiddelbart ble nærmest en **fyrlykt** i taket skudd på. Han tenkte på om han skulle feie garasjegulvet igjen, der hvor han hadde forlatt kosten. Per var dekket i et **gjennomskinnelig lys**. Han begynte å bevege seg mot trappen, hans høyre hånd holdt godt fast i rekkverket til trappen. Han begynte å gå ned den **opplyste** trappen. Hans mål var å finne kosten igjen og fortsette rengjøringen. Hele interiøret hans var oversvømt i de **skimrende strålene** fra taket. Han bevegde seg gjennom de samme rommene han hadde gått gjennom tidligere da han lette etter sikringsboksen. Døren til garasjen var fortsatt åpen, og **gløden** fra **lampene** var synlig helt ut i gangen. Han gikk inn i garasjen og plukket opp kosten igjen. Den intense **gylne, glansen** i rommet var akkurat som før. Per forstod at han følte seg trøtt og bestemte seg for å legge ned kosten.

#5 tidsstempel: stopp måling av pupillediameter

Fortelling 3

Tidsstempel 1: start måling av pupillediameter

Nøytralt avsnitt, 150 ord, 0 målord.

Karis sykkel trillet fremover gjennom gatene. Hun syklet gjennom byen, bena hennes gikk opp og ned i en naturlig rytme. Det kom en svak summing fra dekkene som rullet over asfalten. Gate etter gate forandret landskapet og omgivelsene seg. Hun la merke til hvordan forskjellige materialer var blitt brukt i bygningene avhengig av når og i hvilken stil de var bygget. Kari passerte noen gamle leilighetsblokker laget av murstein som viste tegn til aldring. Her og der kunne Kari se litt slitasje på mursteinen. Hun fortsatte å sykle. Noen kafeer med lukkede dører dukket av og til opp. Trær var plantet langs fortauet som hun syklet på. Noen av bygningene hun

passerte hadde busker som hadde vokst langt oppover veggene. Stilkene på buskene virket å finne noen små hull i disse veggene å gripe tak i. Kari fortsatte å sykle gjennom gatene og merket seg slike detaljer langs hele veien.

Tidsstempel 2: fortsett måling av pupillediameter

Mørkt avsnitt. 149 ord, 6 sammensatte målord, 10 diskrete målord.

Det var blitt sent og gatene ble raskt omsluttet av **kveldsmørket**. Kari syklet over på en grusvei hvor dekkene hennes nå lagde en annen lyd. Trærne som omringet henne viste seg som **dunkle skygger** på denne veien. Etter en stund nærmet Kari seg en park som var omringet av gjerder. Parken så skjult ut og virket fylt med et **beksvart mørke** som pakket seg rundt trærne og benkene. Kari hørte kvitringen fra sirisser i trærne som vokste rundt parken. Da hun nærmet seg midten av parken ble hun omsluttet av en slags **dyp, sort** masse. Hun skimtet så vidt noen basketballkurver rundt midten av parken. Denne massen som omsluttet henne var tykk og **ugjennomsiktig.** Den la seg rundt henne som et teppe. Hun var pakket inn i et **nedtonet skyggeaktig** stoff som gjorde det vanskelig å se alle de små detaljene rundt henne, hun valgte derfor å forlate parken.

Tidsstempel 3: fortsett måling av pupillediameter

Lyst avsnitt. 152 ord, 6 sammensatte målord, 10 diskrete målord.

Kari syklet videre, rundet et hjørne og oppdaget plutselig noe uventet. En **opplyst, skinnende** søyle stod alene på en liten gressflekk foran henne. Den var stor og høy og når hun syklet nærmere så hun at den var laget av stein. Den **gnistrende belysningen** var montert mange steder på søylen, og også ved siden av den på plenen. Hun hoppet av sykkelen, grep rattet og styrte sykkelen fremover mens hun gikk. Søylen **strålte** virkelig veldig sterkt. Fortsatt med sykkelen i hendene gikk hun nærmere søylen. Hun så opp på det **skimrende lyset** som omkranset søylen. Hun la sykkelen fra seg og fortsatte deretter å bevege seg framover og merket seg gresset under føttene hennes. Hun nådde helt inntil søylen og var helt dekket i en **glødende klarhet**. Steinen hadde mønstre og merker etter å ha blitt formet og arbeidet med. Kari så at krystaller i steinen **glinset** når hun så helt nærme.

Tidsstempel 4: fortsett måling av pupillediameter

Mørkt avsnitt. 150 ord, 6 sammensatte målord, 10 diskrete målord.

Kari bestemte seg for å forlate stedet, plukket opp sykkelen og dro av sted. På ny la **nattens mørke** seg raskt rundt henne. Igjen syklet hun på en grusvei, og lyden av sykkelhjulene hennes fylte luften igjen. Kari var nå ute i den **formørkede dysterheten** til den åpne verden. Luften var kjøligere og skarpere i dette miljøet. Trærne og konturene av landskapet rundt var bare synlige som **dempede skygger**. Veien hun syklet på strakk seg flatt framover, mens det på hennes høyre side var en liten høyde. Hun syklet under noen trær og så opp på et helt **beksvart** tak. Kari så at bladene i trærne skapte et tak over henne som dekket utsikten. Noen fugler fløy forbi henne og fylte denne **uklare midnatten** med noen lyder i tillegg til hjulene som trillet over grusen. Noen ugler ga lyd fra seg ikke langt unna. Bare **nattaktive** dyr var fortsatt våkne.

Tidsstempel 5: stopp måling av pupillediameter

Fortelling 4

#1 tidsstempel: start måling av pupillediameter

Nøytralt avsnitt. 151 ord, 0 målord.

Anne forberedte seg for en gåtur med en strukturert rutine. Hun startet rutinen med å velge sine vanlige og slitte, men fortsatt funksjonelle joggesko, og knyttet lissene på dem for en presis passform. Så hentet hun genseren

sin fra kroken på døren, tok den på seg og justerte ermene til de satt riktig. Hun stod i døråpningen, åpnet døren og inhalerte. Hun forsøkte å kjenne duftene som fantes utenfor huset hennes. Hun justerte hetten litt for å være sikker på at den passet på hodet hennes. Hun kikket rundt i gangen sin. Hun kjente at luft fra utsiden bevegde seg innover i huset. Anne forsikret seg om at nøklene hennes var i lommene på genseren. Hun grep ned i høyre lomme for også å forsikre seg om at hun hadde tatt med seg lommeboken. Hun gikk ut og ble stående på dørmatten foran inngangsdøren. Hun snudde seg rundt og lukket døren.

#2 tidsstempel: fortsett måling av pupillediameter

Mørkt avsnitt. 150 ord, 6 sammensatte målord, 10 diskrete målord.

Anne snudde seg rundt igjen, så mot veien fra huset sitt og oppdaget at det var **nattsvart** ute. Luften var skarp og fylt med duften av jord. Hun ble omfavnet av den **dunkle** og **dempede** atmosfæren i verden utenfor hjemmet sitt. Hun startet å gå over grusveien mot hovedveien. Atmosfæren var en **nattlig dunkelhet** som fylte hele synet hennes. Hun hørte mange forskjellige lyder rundt seg. Den **uklare svartheten** som også var rundt henne virket å forsterke naturens lyder. Hun kom til hovedveien hvor underlaget hun gikk på endret seg. Ingen andre var i sikte, bare hun og det **skyggeleggende mørket** var ute. Hun kunne høre skoene sine lage lyd på den harde overflaten. Omgivelsene var **nedtonede** nå som Anne kom nærmere der hun ville ta av hovedveien og inn på en sti til høyre. Hun gikk i et rolig tempo mens hun tenkte og lyttet til lydene rundt henne.

#3 tidsstempel: fortsett måling av pupillediameter

Lyst avsnitt. 150 ord, 6 sammensatte målord, 10 diskrete målord.

Alt var stille helt til **frontlysene** fra en bil plutselig dukket opp i det fjerne. Anne fortsatte å gå vel vitende om at hun var nær stien hun ville ta av på. To **blendende lysstråler** brøt gjennom landskapet et godt stykke foran henne. Anne tok hendene i lommene på genseren sin og fortsatte å gå. Bilens **glødende lyskilde** kom raskt nærmere fra det fjerne. Anne kunne nå se starten på stien som gikk til høyre. Gåturen hennes ble avbrutt av de to **lyskasterne** fra bilen. Stien var nå bare femti meter foran henne, hun fortsatte å nærme seg den. De kraftige, **gnistrende strålene** traff henne rett i øynene mens bilen nærmet seg. Anne hadde en liten papirlapp i lommen som fingrene hennes begynte å leke med. Den **skinnende klarheten** fra bilen hvilte over hele henne idet den kjørte forbi. Et vindpust traff henne rett før hun tok av på stien.

#4 tidsstempel: fortsett måling av pupillediameter

Mørkt avsnitt. 146 ord, 6 sammensatte målord, 10 diskrete målord.

Anne var igjen alene og merket den plutselige tilbakekomsten av den **slukkede skyggeverdenen** hun hadde befunnet seg i tidligere. Den plutselige overgangen gjorde henne nesten desorientert, men føttene hennes fortsatte allikevel å bevege seg. Omgivelsene hennes var igjen **mørklagte**, landskapet lignet det fra tidligere. Høyresvingen inn på stien ledet henne til den grusveien hun hadde planlagt for. De **mørke skyggene** av fugler og trær krøp sakte tilbake til sine kjente plasser. Hun fortsatte i et jevnt tempo. Den **bekmørke, ugjennomsiktige** atmosfæren fra tidligere var rundt henne. Anne tok hendene ut av lommene og lot dem svinge i koordinasjon med føttene. Den **dunkle, kullsvarte** konturen av stien foran henne ledet veien fremover. Hun fortsatte turen sin på grusveien. Det var nå bare de **sorte** konturene av tingene rundt henne som kunne skjelnes. Bekken nær hjemmet hennes kunne høres og hun visste at hennes gåtur snart var ferdig.

#5 tidsstempel: stopp måling av pupillediameter

<u>Post-experimental questionnaire</u>

<u>Original</u>

Spørsmål til deltakernes hukommelse av historiene.

1. I fortellingen med Per som kostet huset, gikk Per inn igjen i garasjen etter lysene var skrudd på igjen? **Ja**
2. I fortellingen med Anne som gikk på tur, tok Anne av på en sti som gikk til venstre? **Nei**
3. I fortellingen med Ole som var på biltur, kjørte Ole forbi en stor, rød bondegård? **Nei**
4. I fortellingen med Kari som syklet, skimtet Kari noen basketballkurver i parken hun syklet inn i? **Ja**

---- Sideskift ----

Spørsmål om narrativ absorpsjon og om skapelsen av mentale bilder:

I hvor stor grad ble du **absorbert** inn i historien med Per som kostet?

Mens jeg leste fortellingen om Per med kosten, kunne jeg levende forestille meg Per. (Ordvalg basert på Green & Brock (2000: 704) sine items for Transportation scale)

I hvor stor grad stemmer følgende påstand:

1. Mens jeg leste fortellingen om Per med kosten, kunne jeg levende forestille meg Per.
2. Mens jeg leste fortellingen om Anne som gikk på tur, kunne jeg levende forestille meg Anne.
3. Mens jeg leste fortellingen om Ole som var på biltur, kunne jeg levende forestille meg Ole.
4. Mens jeg leste fortellingen om Kari som syklet, kunne jeg levende forestille meg Kari.

1 = Ikke I det hele tatt --2-- --3-- --4-- --5-- --6-- 7 = Veldig mye

---- Sideskift ----

Om spenning i historien:

1. I hvor stor grad følte du spenning i historien om Per som kostet i huset?
2. I hvor stor grad følte du spenning i historien om Anne som gikk på tur?
3. I hvor stor grad følte du spenning i historien om Ole som var på biltur?
4. I hvor stor grad følte du spenning i historien om Kari som syklet?

1 = Ikke I det hele tatt --2-- --3-- --4-- --5-- --6-- -- 7 = Veldig mye

English version

Attention questions (correct answer marked in bold), yes/no answers

1. In the story about Per who broomed the house, did Per og back into the garage after the lights were turned on again? Yes
2. In the story with Anne who went for a walk, did Anne take a path that went to the left? No
3. In the story with Ole who was on a road trip, did Ole drive past a big, red farmhouse? No
4. In the story with Kari who was cycling, did Kari glimpse some basketball hoops in the park she rode into? Yes

---- Page break ----

To what extent do you agree with the following statements:

1. While reading the story about Per with the broom, I could vividly imagine Per.
2. While reading the story about Anne who went for a walk, I could vividly imagine Anne.
3. While reading the story about Ole who was on a road trip, I could vividly imagine Ole.
4. While reading the story about Kari who was cycling, I could vividly imagine Kari.

Scale: 1 = Not at all --2-- --3-- --4-- --5-- --6-- 7 = Very much

---- Page break ----

1. To what extent did you feel excitement in the story about Per sweeping in the house?
2. To what extent did you feel excitement in the story about Anne who went for a walk?
3. To what extent did you feel excitement in the story about Ole who was on a road trip?

4. To what extent did you feel excitement in the story about Kari who was cycling?

Scale: 1 = Not at all --2-- --3-- --4-- --5-- --6-- 7 = Very much

---

Experiment 2

Stimuli (written version)

lang	cond	text
en	bright_happy	That day, I was gathering with my friends and family in a luxurious garden for my birthday celebration. The sun was shining brightly, as a vibrant blue sky stretched overhead. A small pond nearby reflected the sunlight, casting sparkles of light on the surrounding foliage. As I chatted with my loved ones, a sudden burst of excitement filled the air. In unison, they started singing a birthday song, holding a strawberry cake adorned with an abundance of candles. The sunlight sparkled on the frosting as they approached. With a beaming smile, I made my wish and blew out the candles, surrounded by cheers and applause.
nl	bright_happy	Die middag vierde ik met vrienden en familie mijn verjaardag in een luxueuze tuin. De zon scheen fel, en de lucht was helder blauw. Zonlicht weerkaatste van een kleine vijver in de buurt, en de reflectie scheen op het omliggende gebladerte. Terwijl ik met mijn vrienden en familie praatte, merkte ik opeens dat er een plezierige spanning in de lucht hing. In koor begon iedereen een verjaardagslied te zingen, terwijl ze een aardbeientaart tevoorschijn haalden die versierd was met een overvloed aan kaarsen. Het zonlicht glinsterde op het glazuur van de taart terwijl ze de taart naar me toe brachten. Met een stralende glimlach deed ik een wens en blies de kaarsen uit, omringd door gejuich en applaus.
en	dark_happy	That evening, I was gathering with my friends and family in my comfortable living room for my birthday celebration. As I was chatting to my loved ones, a sudden burst of excitement filled the air. The lights went out, plunging the space into darkness. I saw a cake decorated with a multitude of candles coming towards me, and my friends began to sing a birthday song. I couldn't stop smiling. The room was lit only by the soft, warm light of the candles, creating an intimate and magical atmosphere. As I made my wish and blew out the candles, the room erupted in cheers and applause.
nl	dark_happy	Die avond vierde ik met vrienden en familie mijn verjaardag in een comfortabele woonkamer. Terwijl ik met mijn vrienden en familie praatte, merkte ik opeens dat er een plezierige spanning in de lucht hing. De lichten gingen uit en de ruimte werd gedompeld in duisternis. Ik zag een taart met een heleboel kaarsjes op me afkomen terwijl mijn vrienden een verjaardagsliedje begonnen te zingen. Ik kon niet stoppen met glimlachen. De kamer werd slechts verlicht door het zachte, warme licht van de kaarsen, wat een intieme en magische sfeer creëerde. Terwijl ik een wens deed en de kaarsen uitblies, barstte iedereen uit in gejuich en applaus.

en	bright_neutral	Judging by the sun in the sky, it was nearly lunchtime when I woke up this morning. So I got out of my tent and walked along the riverbank. The sun's rays reflected off the surface of the lake and blinded me. I'd forgotten my sunglasses. Looking up at the cloudless blue sky, I realised that it wouldn't be raining for a long time. So I grabbed my cap to protect myself from the sun and carried on walking.
nl	bright_neutral	Gezien de stand van de zon aan de hemel, was het al bijna lunchtijd toen ik wakker werd. Dus stapte ik uit mijn tent en liep langs de rivieroever. De zonnestralen weerkaatsten op het oppervlak van het water en verblindden me. Ik was mijn zonnebril vergeten. Terwijl ik naar de wolkeloze blauwe lucht keek, besefte ik me dat het voorlopig niet zou gaan regenen. Dus zette ik een pet op om mezelf tegen de zon te beschermen en liep verder.
en	dark_neutral	It was already nightfall when I got home. There was no electricity because of the storm, so I ventured into the living room in search of candles. It was very dark, but I knew the place by heart. I went straight to the cupboard and took out a candle. After lighting it, I made myself a hot chocolate by the flickering light of the candle and stood there, watching the storm through the window.
nl	dark_neutral	De avond begon al te vallen toen ik thuis kwam. Er was geen elektriciteit vanwege de storm, dus ging ik naar de woonkamer op zoek naar kaarsen. Het was erg donker, maar ik wist waar ik moest zoeken. Ik ging rechtstreeks naar de kast en pakte een kaars. Nadat ik hem had aangestoken, maakte ik een warme chocolademelk voor mezelf bij het flakkerende licht van de kaars en stond daar, kijkend naar de storm door het raam.
en	bright_to_dark	The room I entered was enveloped in an intense, blinding light, obscuring everything in its brilliance. As I took a moment to adjust, a radiant source of light shone vividly right in front of me. Driven by curiosity, I reached out to touch it, but as my hand extended, the light began to drift apart, distancing itself from me. It receded to a point so far away that all I could do was watch as it grew smaller and dimmer. Eventually, the brilliance faded into an inky darkness, rendering the room pitch black, so dark that I couldn't see a thing.
nl	bright_to_dark	In de kamer die ik binnenging was er een in intense, verblindende lichtbron die alles met zijn glans bedekte. Terwijl ik een moment nam om hieraan te wennen, straalde een stralende lichtbron levendig vlak voor me. Uit nieuwsgierigheid probeerde ik de lichtbron aan te raken, maar toen ik mijn hand uitstrekte, begon het licht op te lossen en zich van mij te verwijderen. De lichtbron trok zich terug naar een punt dat zo ver weg was dat ik er alleen nog maar naar kon kijken, terwijl het kleiner en zwakker werd. Uiteindelijk vervaagde de glans tot een inktzwarte duisternis overbleef, waardoor de kamer pikdonker werd, zo donker dat ik niets meer kon zien.

en	dark_to_bright	The room I walked in was so dark that I couldn't see a thing, it was pitch black. After a moment, my eyes adjusted to the darkness, and I could see a very tiny, small source of light far away in front of me. So I decided to walk in that direction, right towards the light. As I approached, the light was growing stronger and stronger, growing more luminous with each passing second. With every step, it appeared closer to me. At some point, the light was so bright that I couldn't see anything else, the room was enveloped in an intense, blinding light.
nl	dark_to_bright	In de kamer die ik binnenging was het zo donker dat ik helemaal niets kon zien, het was pikdonker. Na een tijdje wenden mijn ogen aan de duisternis, en zag ik in de verte een zeer kleine, kleine lichtbron. Dus ik besloot ernaartoe te lopen, recht naar het licht. Naarmate ik dichterbij kwam, werd het licht steeds sterker en helderder. Met elke voorbijgaande seconde leek het meer licht te geven en dichterbij te komen. Op een gegeven moment was het licht zo fel dat ik niets anders meer kon zien. De kamer baadde in een intens, verblindend licht.
en	dark_lotr	As darkness settled over the Shire like a soft blanket, Bilbo's hobbit hole nestled comfortably into the hillside, its windows emitting a gentle glow from the warm light of lanterns within. Stepping inside, I found myself enveloped in a calm atmosphere, the dimness casting familiar objects in a soft, shadowy embrace. The crackling of embers from the fireplace provided a soothing soundtrack to the scene, while the flickering candles scattered throughout the room added to the cosy ambiance.
nl	dark_lotr	Terwijl duisternis zich als een zachte deken over de Gouw verspreidde, nestelde Bilbo's hobbitgat zich comfortabel in de heuvelwand, waarvan de ramen een zachte gloed uitstraalden van het warme licht van lantaarns binnen. Toen ik naar binnen stapte, voelde ik me omhuld door een rustige sfeer, waarin het schemerige licht vertrouwde objecten in een zachte, schaduwrijke omhelzing plaatste. Het knisperen van de smeulende kolen in de open haard zorgde voor een rustgevend geluid, terwijl de flikkerende kaarsen verspreid door de kamer bijdroegen aan de gezellige sfeer.
en	bright_lotr	In the radiant splendour of Rivendell's Elven gardens on a sun-kissed day, I found myself surrounded by a symphony of nature's finest delights. Each step along the winding paths revealed a new burst of colour as vibrant blooms danced in the sunlight, their petals aglow with warmth. Butterflies fluttered gracefully amidst the foliage, their elegant flight adding a touch of magic to the scene. As I strolled beside the babbling brook, the gentle murmur of water provided a peaceful backdrop to this calm oasis.
nl	bright_lotr	In de stralende pracht van de Elfentuinen van Rivendell op een door de zon gekuste dag, bevond ik mezelf omringd door een symfonie van de fijnste geneugten van de natuur. Elke stap langs de kronkelende paden onthulde een nieuwe explosie van kleuren terwijl levendige bloesems dansten in het zonlicht, hun bloemblaadjes gloeiden van warmte. Vlinders fladderden sierlijk te midden van het gebladerte, een vleugje magie toevoegend aan het tafereel met hun elegante vleugels. Terwijl ik langs de kabbelende beek wandelde, zorgde het zachte geruis van het water voor een vredige achtergrond bij de rustige oase.

Experiment 3

Instructions provided to narrator

General instructions

- The stories should be read like you are telling a children's tale, slow enough and with pauses between sentences so that the listener has time to picture the story in his/her mind.

Each story version is divided in two parts, a bright and a dark part (V1: bright then dark; V2: dark then bright).

- Each part should be read as similarly as possible, in terms of emotions conveyed, intonation, pace, duration, volume, etc. so that the only thing that differs between the two parts is the brightness level of the mental images that come to mind while people are listening to it.

Specific instructions

- Stories should ideally be read slowly enough so that non-native English speakers have enough time to process the meaning of the story and imagine its content.
- Each story part (bright and dark) should ideally have the same duration

Stimuli (written version)

STORY V1 – First Bright, then Dark

Bright: 313 words – Dark: 312 words

I had the most amazing dream the other day... It was a bright, sunny day. One of those days in the middle of summer when everyone is out enjoying the weather and feeling the warm sun on their skin and faces. I was lying in the grass with my eyes open, looking at the bright blue sky above me. I was surrounded by light. I put my hand up to shield my eyes from the sun, but the overwhelming brightness still made me squint. It was surreal, all that brightness. It was like everything was covered in glitter. Bright, sparkling glitter. I played with the sun's rays, bending them, making them reflect bright colours off the shiny grass. It was as if all the colours of the rainbow were dancing together in a symphony of light. Suddenly I heard little feet tapping on the grass beside me. Tippity-tappety. I sat up to see where the sound was coming from. Tippity-tappety again. A fluffy white rabbit was sitting on the grass beside me. It was looking at me with two big round eyes. Its fur was so white that it shone like the sun itself. Sitting in the sunlight, more sparkling than ever, I realised it must be the Great Rabbit of Light. "Look," the rabbit said, pointing up at the sky towards the sun. Without thinking, I looked up, only to be blinded by the incredible brightness of the sun. "It's all right," said the rabbit. "It's just the sun. She's a friend. Look how bright and beautiful she is." I forced myself to keep looking, and indeed she was, so very bright and so very pretty. Then I looked down again. It took a few moments for my vision to clear and for me to see the rabbit again, waiting patiently until I was ready. It wanted to show me something else. …

[leave a small break]

The rabbit had waited patiently. "Close your eyes," it said, "I want to show you something.” I did as the rabbit said and closed my eyes. At first I could still see the faint redness of the light penetrating my eyelids. But then the red began to fade and it became dark, a pitch black darkness. "Now open your eyes," said the rabbit. Again I did as the rabbit told me. Night had fallen. But it was a special kind of night, because everything around me was silent and peaceful. At first I couldn't see anything, as if I were in a completely dark room. Slowly my eyes adjusted to the darkness and a nocturnal world of subtle shades of grey and black appeared. Suddenly I heard the tapping of small feet on the grass beside me. Tippity tappety. I knew that sound. Tippity-tappety again. Standing in the shadows, the rabbit had turned into night and had become the Great Rabbit of Darkness. It was sitting next to me, surrounded by little fireflies that circled its head, casting a faint glow over its dark fur. I looked up at the sky and saw no stars, because they were hidden behind thick layers of clouds that had suddenly appeared and kept the world in darkness. The dark grass seemed to stretch out behind the rabbit, but it was too dark for me to see far. It was a symphony of shadows. "It's OK," the rabbit said. "Take in the magical night. She’s

a friend too. Let the darkness flow through you". And once again I did as the rabbit said and surrendered to the shadows. It was magical. And as I stood there, wrapped in the embrace of darkness, I knew that this moment would stay with me long after the night had faded into dawn. "Congratulations," the rabbit said. "You have completed your journey."

Audio version truncated to 1:57:500 to make sure both story parts have the same duration (excludes the highlighted text)

STORY V2 – First Dark, then Bright

Bright: 309 words Dark: 310 words

I had the most amazing dream the other night... It was night. But it was a special kind of night, everything around me was silent and peaceful. At first it was so dark, a pitch black darkness, that I couldn't see a thing. It was like being in a completely dark room. Slowly, my eyes adjusted to the darkness and a nocturnal world of subtle shades of grey and black appeared. I knew I was dreaming, but it was still amazing. Suddenly, I heard the tapping of small feet on the grass beside me. Tippity-tappety. I looked around to see where the sound was coming from. Tippity-tappety again. Standing in the shade beside me, I saw a fluffy black rabbit. Its fur was so dark in the deepness of the night that it seemed to be wearing the night itself. It was staring at me, surrounded by little fireflies that were circling his head, casting a faint glow over its dark fur. No doubt. It must be the Great Rabbit of Darkness. I looked up at the sky and saw no stars, for they were hidden behind thick layers of cloud that had suddenly appeared and kept the world in darkness. The dark grass seemed to stretch out behind the rabbit, but it was too dark for me to see far. It was a symphony of shadows, so dark. "It's OK," the rabbit said. "Take in the magical night. She's a friend. Let the darkness flow through you." And once again I did as the rabbit said and surrendered to the shadows. It was magical. And as I stood there, wrapped in the embrace of darkness, I knew that this moment would stay with me long after the night had faded into dawn. "When you are ready, follow the fireflies," the rabbit said, "I want to show you something else." …

[leave a small break]

I had followed the fireflies and the night had turned into day. It was a bright, sunny day. One of those days in the middle of summer when everyone is out enjoying the weather and feeling the warm sun on their skin and faces. I was lying in the grass with my eyes open, taking in the bright blue sky above me. I was surrounded by light. I put my hand up to shield my eyes from the sun, but the overwhelming brightness still made me squint. It was surreal, all the brightness. I knew I was still dreaming because it was as if everything was covered in glitter. Bright, sparkling glitter. I played with the sun's rays, bending them, making them reflect bright colours off the shiny grass. It was as if all the colours of the rainbow were dancing together in a symphony of light. Suddenly I heard little feet tapping on the grass beside me. Tippity-tappety. I knew that sound. Tippity-tappety again. This time, a fluffy white rabbit was sitting on the grass beside me, looking at me with two big round eyes. Its fur was so white that it glowed like the sun himself. I realised that the rabbit had transformed into the Great Rabbit of Light. And there it was, sitting in the sunlight, more sparkling than ever. "Look," the rabbit said, pointing up at the sky towards the sun. Without thinking, I looked up, only to be blinded by the incredible brightness of the sun. "It's OK," said the rabbit. "It's just the sun. She's a friend too. Look how bright and beautiful she is." I forced myself to keep looking, and indeed she was, so very bright and so very pretty… Then I looked down again and saw the rabbit, still staring at me. "Congratulations," it said. "You have completed your journey".

Audio version truncated to 02:05:500 to make sure both story parts have the same duration (excludes the highlighted text)

ADDITIONAL MATERIALS

EXPERIMENT SLIDES

Experiment 1

POST-EXPERIMENTAL QUESTIONNAIRE

Experiment 2 & 3

Ole våknet opp for bilturen som han hadde planlagt i flere dager. Han gikk ut av sengen og ned trappene i huset sitt, med hendene foldet sammen foran seg. Nede i stuen ved kjøkkenet forberedte han seg for turen ved å følge sine vanlige rutiner. Han så gjennom pakkelisten sin igjen og forsikret seg om at alle essensielle gjenstander var inkludert. Han markerte hver gjenstand som var skrevet på listen med et kryss. Ifølge listen skulle alt være pakket og lastet i bilen. Ole gikk til badet sitt, pusset tenner og fant toalettmappen sin hvor han kunne pakke sine siste gjenstander. Han pleide alltid å glemme noe, men ikke denne gangen. Med alt pakket og alle forberedelser fullført låste Ole hjemmet sitt. Han gikk til garasjen og åpnet den elektriske porten med en fjernkontroll. Han åpnet fordøren til bilen sin, satte seg inn og skrudde på motoren. ->

Det var en strålende sommerdag da Ole kjørte på motorveien i bilen sin. Alt var klart for en tur som skulle vare i flere dager. Solstrålene skinte på bilpanseret mens han kjørte. Det nærmet seg flere svinger i veien foran ham og Ole bestemte seg av den grunn for å skifte gir med sin høyre hånd. Asfalten han kjørte på skimret i varmen foran ham. Ole kunne nå se fjerne fjell i horisonten. Det blendende lyset gjorde kjøringen gjennom svingene vanskeligere, men Ole var stødig bak rattet og kjørte kontrollert. Han hadde sin venstre hånd på rattet og hvilte ryggen sin mot bilsetet. Landskapet rundt ham nærmest glinset mens Ole fortsatte kjøringen og kom enda nærmere fjellene han så foran seg. Store steiner som lå oppover fjellene begynte å bli synlige mens Ole åpnet vinduet sitt for å slippe luft inn i bilen. Dette var absolutt en varm og solfylt dag. ->

Turen fortsatte og motorveien førte Ole videre mot et stort fjell og en mørk tunnel gjennom det. Ole plasserte sin høyre hånd på rattet og brukte sin venstre hånd for å lukke vinduet. Temperaturen sank da han nærmet seg den sorte inngangen. Veien gikk som en rett strek rett framover. Da Ole kjørte gjennom inngangen, begynte svarte skygger å dukke opp. Han passerte under to store vifter som lagde en dyp lyd som forsvant da han passerte dem. Ole kjørte gjennom en ugjennomtrengelig bekmørk natt. Han kjente gasspedalen under høyre fot og trykket den litt ned. Dette dunkle skyggeområdet var som en ny verden for ham. Ole trykket clutchen helt ned for å gjøre seg klar til å skifte gir med høyre hånd. Veggene rundt ham var dekket av et kullsvart lag med gammel eksos. Motoren ga fra seg en høyere tone mens veien begynte å gå oppover. ->

Skilt langs veien indikerte at utgangen var nær, og Ole kunne se det klare lyset nærme seg i enden. Veien fortsatte enda i en rett linje framover, slik den hadde gjort en stund. Den strålende, hvite utgangen ble større ettersom Ole nærmet seg den. Han skiftet gir og akselererte, nålen på speedometeret steg sakte. I det øyeblikket Ole kjørte gjennom utgangen, ble han overveldet av blendende dagslys på den andre siden. Først var det vanskelig å se, men etter hvert begynte konturene i omgivelsene å dukke opp igjen. Det sterke sollyset la seg over hele landskapet og også inn i bilen til Ole. Han begynte å se trær og store steiner på fjellene rundt ham. Toppene på de fjerne fjellene var badet i en tydelig gyllen glød. Trærne var høye med greiner på toppen, og steinene var store og runde. Ole hadde fortsatt flere timer igjen å kjøre i disse glitrende omgivelsene. ->

Per dro kosten over teppet. Huset trengte å bli rengjort. Han fortsatte å feie gulvet mens han beveget seg fra stuen til gangen. Han feide smuler og dyrehår opp fra gulvet til et feiebrett. Lyden av kosten mot teppet ga fra seg en rytmisk lyd. Han flyttet seg til kjøkkenet, hvor han bevegde seg rundt spisebordet og stolene. Noen magasiner lå på den ene enden av bordet, og alle stolene hadde puter på seg som han nettopp hadde kjøpt i butikken. Per ble ferdig i dette rommet og bevegde seg mot garasjen med kosten fortsatt i hendene sine. Han passerte en stabel uåpnet post som lå på kjøkkenbenken. Inni garasjen var det rotete. Hyller, bokser og verktøy var rundt omkring. En stor verktøykasse med en hammer, noen skruer og mange forskjellige andre ting, sto åpen på gulvet. Han stoppet et sekund og så bort på de støvete hyllene og gulvet. ->

Den skinnende belysningen som kom fra taket i garasjen gjorde hver eneste tomme av rommet synlig. Per feide også her støv og diverse småstein fra gulvet til feiebrettet. De sterke spotlysene ovenfra ble reflektert fra de metalliske overflatene av stativene hvor verktøy og utstyr lå. Verktøyet ble ikke ofte brukt, men det hadde allikevel vært veldig praktisk å ha fra tid til annen. Lampenes opplysning dekket hele rommet. Flere bokser var plassert oppå hverandre i et hjørne av garasjen. I denne nesten klinisk, hvite klarheten var det mulig for Per å feie i de små glipene som fantes mellom disse boksene. Han snudde seg mot hyllene og feide disse også. Den intense lyskilden nådde til og med ned til de laveste hyllene lengst ned mot gulvet. Lyden fra kosten fortsatte mens Per rengjorde rundt i garasjen. Den strålende lysstyrken i rommet fortsatte også å være til stede mens rengjøringen pågikk. ->

Plutselig spredte en stillhet seg rundt Per. Garasjen stupte inn i et ugjennomsiktig mørke. Han stoppet rengjøringen og sto stille, hans hånd grep fortsatt rundt kosteskaftet. Han var omgitt av en tykk, beksvart masse. Elektrisiteten var borte. Garasjen og alle hyllene ble fortært av denne tykke svartheten. Han måtte finne sikringsboksen og slå på hovedbryteren. Etter noen sekunder begynte han å gå gjennom det dystre mørket mot der han tenkte døren inn til hjemmet hans var. Etter noen skritt var døren foran ham, og han fant dørhåndtaket og trykket det ned. På den andre siden kunne han skimte dunkle skygger av interiøret hans i stuen, som sofaen, stolene og bordet. Sikringsboksen var ved toppen av trappen, og Per begynte å føle seg fram gjennom gangen mot trappen. Han fant trappen og snublet seg oppover i denne mørke natten og nådde toppen. Han åpnet sikringsboksen og fant hovedbryteren. ->

Fingrene hans vippet opp hovedbryteren, og umiddelbart ble nærmest en fyrlykt i taket skudd på. Han tenkte på om han skulle feie garasjegulvet igjen, der hvor han hadde forlatt kosten. Per var dekket i et gjennomskinnelig lys. Han begynte å bevege seg mot trappen, hans høyre hånd holdt godt fast i rekkverket til trappen. Han begynte å gå ned den opplyste trappen. Hans mål var å finne kosten igjen og fortsette rengjøringen. Hele interiøret hans var oversvømt i de skimrende strålene fra taket. Han bevegde seg gjennom de samme rommene han hadde gått gjennom tidligere da han lette etter sikringsboksen. Døren til garasjen var fortsatt åpen, og gløden fra lampene var synlig helt ut i gangen. Han gikk inn i garasjen og plukket opp kosten igjen. Den intense gylne, glansen i rommet var akkurat som før. Per forstod at han følte seg trøtt og bestemte seg for å legge ned kosten. ->

Karis sykkel trillet fremover gjennom gatene. Hun syklet gjennom byen, bena hennes gikk opp og ned i en naturlig rytme. Det kom en svak summing fra dekkene som rullet over asfalten. Gate etter gate forandret landskapet og omgivelsene seg. Hun la merke til hvordan forskjellige materialer var blitt brukt i bygningene avhengig av når og i hvilken stil de var bygget. Kari passerte noen gamle leilighetsblokker laget av murstein som viste tegn til aldring. Her og der kunne Kari se litt slitasje på mursteinen. Hun fortsatte å sykle. Noen kafeer med lukkede dører dukket av og til opp. Trær var plantet langs fortauet som hun syklet på. Noen av bygningene hun passerte hadde busker som hadde vokst langt oppover veggene. Stilkene på buskene virket å finne noen små hull i disse veggene å gripe tak i. Kari fortsatte å sykle gjennom gatene og merket seg slike detaljer langs hele veien. ->

Det var blitt sent og gatene ble raskt omsluttet av kveldsmørket. Kari syklet over på en grusvei hvor dekkene hennes nå lagde en annen lyd. Trærne som omringet henne viste seg som dunkle skygger på denne veien. Etter en stund nærmet Kari seg en park som var omringet av gjerder. Parken så skjult ut og virket fylt med et beksvart mørke som pakket seg rundt trærne og benkene. Kari hørte kvitringen fra sirisser i trærne som vokste rundt parken. Da hun nærmet seg midten av parken ble hun omsluttet av en slags dyp, sort masse. Hun skimtet så vidt noen basketballkurver rundt midten av parken. Denne massen som omsluttet henne var tykk og ugjennomsiktig. Den la seg rundt henne som et teppe. Hun var pakket inn i et nedtonet skyggeaktig stoff som gjorde det vanskelig å se alle de små detaljene rundt henne, hun valgte derfor å forlate parken. ->

Kari syklet videre, rundet et hjørne og oppdaget plutselig noe uventet. En opplyst, skinnende søyle stod alene på en liten gressflekk foran henne. Den var stor og høy og når hun syklet nærmere så hun at den var laget av stein. Den gnistrende belysningen var montert mange steder på søylen, og også ved siden av den på plenen. Hun hoppet av sykkelen, grep rattet og styrte sykkelen fremover mens hun gikk. Søylen strålte virkelig veldig sterkt. Fortsatt med sykkelen i hendene gikk hun nærmere søylen. Hun så opp på det skimrende lyset som omkranset søylen. Hun la sykkelen fra seg og fortsatte deretter å bevege seg framover og merket seg gresset under føttene hennes. Hun nådde helt inntil søylen og var helt dekket i en glødende klarhet. Steinen hadde mønstre og merker etter å ha blitt formet og arbeidet med. Kari så at krystaller i steinen glinset når hun så helt nærme. ->

Kari bestemte seg for å forlate stedet, plukket opp sykkelen og dro av sted. På ny la nattens mørke seg raskt rundt henne. Igjen syklet hun på en grusvei, og lyden av sykkelhjulene hennes fylte luften igjen. Kari var nå ute i den formørkede dysterheten til den åpne verden. Luften var kjøligere og skarpere i dette miljøet. Trærne og konturene av landskapet rundt var bare synlige som dempede skygger. Veien hun syklet på strakk seg flatt framover, mens det på hennes høyre side var en liten høyde. Hun syklet under noen trær og så opp på et helt beksvart tak. Kari så at bladene i trærne skapte et tak over henne som dekket utsikten. Noen fugler fløy forbi henne og fylte denne uklare midnatten med noen lyder i tillegg til hjulene som trillet over grusen. Noen ugler ga lyd fra seg ikke langt unna. Bare nattaktive dyr var fortsatt våkne. ->

Anne forberedte seg for en gåtur med en strukturert rutine. Hun startet rutinen med å velge sine vanlige og slitte, men fortsatt funksjonelle joggesko, og knyttet lissene på dem for en presis passform. Så hentet hun genseren sin fra kroken på døren, tok den på seg og justerte ermene til de satt riktig. Hun stod i døråpningen, åpnet døren og inhalerte. Hun forsøkte å kjenne duftene som fantes utenfor huset hennes. Hun justerte hetten litt for å være sikker på at den passet på hodet hennes. Hun kikket rundt i gangen sin. Hun kjente at luft fra utsiden bevegde seg innover i huset. Anne forsikret seg om at nøklene hennes var i lommene på genseren. Hun grep ned i høyre lomme for også å forsikre seg om at hun hadde tatt med seg lommeboken. Hun gikk ut og ble stående på dørmatten foran inngangsdøren. Hun snudde seg rundt og lukket døren. ->

Anne snudde seg rundt igjen, så mot veien fra huset sitt og oppdaget at det var nattsvart ute. Luften var skarp og fylt med duften av jord. Hun ble omfavnet av den dunkle og dempede atmosfæren i verden utenfor hjemmet sitt. Hun startet å gå over grusveien mot hovedveien. Atmosfæren var en nattlig dunkelhet som fylte hele synet hennes. Hun hørte mange forskjellige lyder rundt seg. Den uklare svartheten som også var rundt henne virket å forsterke naturens lyder. Hun kom til hovedveien hvor underlaget hun gikk på endret seg. Ingen andre var i sikte, bare hun og det skyggeleggende mørket var ute. Hun kunne høre skoene sine lage lyd på den harde overflaten. Omgivelsene var nedtonede nå som Anne kom nærmere der hun ville ta av hovedveien og inn på en sti til høyre. Hun gikk i et rolig tempo mens hun tenkte og lyttet til lydene rundt henne. ->

Alt var stille helt til frontlysene fra en bil plutselig dukket opp i det fjerne. Anne fortsatte å gå vel vitende om at hun var nær stien hun ville ta av på. To blendende lysstråler brøt gjennom landskapet et godt stykke foran henne. Anne tok hendene i lommene på genseren sin og fortsatte å gå. Bilens glødende lyskilde kom raskt nærmere fra det fjerne. Anne kunne nå se starten på stien som gikk til høyre. Gåturen hennes ble avbrutt av de to lyskasterne fra bilen. Stien var nå bare femti meter foran henne, hun fortsatte å nærme seg den. De kraftige, gnistrende strålene traff henne rett i øynene mens bilen nærmet seg. Anne hadde en liten papirlapp i lommen som fingrene hennes begynte å leke med. Den skinnende klarheten fra bilen hvilte over hele henne idet den kjørte forbi. Et vindpust traff henne rett før hun tok av på stien. ->

Anne var igjen alene og merket den plutselige tilbakekomsten av den slukkede skyggeverdenen hun hadde befunnet seg i tidligere. Den plutselige overgangen gjorde henne nesten desorientert, men føttene hennes fortsatte allikevel å bevege seg. Omgivelsene hennes var igjen mørklagte, landskapet lignet det fra tidligere. Høyresvingen inn på stien ledet henne til den grusveien hun hadde planlagt for. De mørke skyggene av fugler og trær krøp sakte tilbake til sine kjente plasser. Hun fortsatte i et jevnt tempo. Den bekmørke, ugjennomsiktige atmosfæren fra tidligere var rundt henne. Anne tok hendene ut av lommene og lot dem svinge i koordinasjon med føttene. Den dunkle, kullsvarte konturen av stien foran henne ledet veien fremover. Hun fortsatte turen sin på grusveien. Det var nå bare de sorte konturene av tingene rundt henne som kunne skjelnes. Bekken nær hjemmet hennes kunne høres og hun visste at hennes gåtur snart var ferdig. ->

LimeSurvey

This questionnaire can be completed in either English or Dutch.

At no point will we ask for your identity nor will we register your IP address. Collected data will be encrypted. All analyses will involve anonymised data only.

Help us understand how you experience your 'inner world'!

Section A: Vividness of Visual Imagery Questionnaire (VVIQ)

For each item on this questionnaire, try to form a visual image, and consider your experience carefully. For any image that you do experience, rate how vivid it is using the five-point scale.

If you do not have a visual image, rate vividness as ‘No image at all, you only “know” that you are thinking of the object’.

Only use ‘Perfectly clear and vivid as real seeing’ for images that are truly as lively and vivid as real seeing.

Please note that there are no right or wrong answers to the questions, and that it is not necessarily desirable to experience imagery or, if you do, to have more vivid imagery.

A1. Think of a relative or friend whom you frequently see (but who is not with you at present) and consider carefully the picture that comes before your mind’s eye.

	No image at all, you only “know” that you are thinking of the object	Vague and dim	Moderately clear and vivid	Clear and reasonably vivid	Perfectly clear and vivid as real seeing
The exact contour of face, head, shoulders and body.	☐	☐	☐	☐	☐
Characteristic poses of head, attitudes of body etc.	☐	☐	☐	☐	☐
The precise carriage, length of step etc, when walking.	☐	☐	☐	☐	☐
The different colours worn in some familiar clothes.	☐	☐	☐	☐	☐

A2. Visualise a rising sun. Consider carefully the picture that comes before your mind’s eye.

	No image at all, you only “know” that you are thinking of the object	Vague and dim	Moderately clear and lively	Clear and reasonably vivid	Perfectly clear and vivid as real seeing
The sun rising above the horizon into a hazy sky.	☐	☐	☐	☐	☐
The sky clears and surrounds the sun with blueness.	☐	☐	☐	☐	☐
Clouds. A storm blows up with flashes of lightning.	☐	☐	☐	☐	☐
A rainbow appears.	☐	☐	☐	☐	☐

A3. Think of the front of a shop which you often go to. Consider the picture that comes before your mind's eye.

	No image at all, you only "know" that you are thinking of the object	Vague and dim	Moderately clear and vivid	Clear and reasonably vivid	Perfectly clear and vivid as real seeing
The overall appearance of the shop from the opposite side of the road.	☐	☐	☐	☐	☐
A window display including colours, shapes and details of individual items for sale.	☐	☐	☐	☐	☐
You are near the entrance. The colour, shape and details of the door.	☐	☐	☐	☐	☐
You enter the shop and go to the counter. The counter assistant serves you. Money changes hands.	☐	☐	☐	☐	☐

A4. Finally think of a country scene which involves trees, mountains and a lake. Consider the picture that comes before your mind's eye.

	No image at all, you only "know" that you are thinking of the object	Vague and dim	Moderately clear and vivid	Clear and reasonably vivid	Perfectly clear and vivid as real seeing
The contours of the landscape.	☐	☐	☐	☐	☐
The colour and shape of the trees.	☐	☐	☐	☐	☐
The colour and shape of the lake.	☐	☐	☐	☐	☐
A strong wind blows on the trees and on the lake causing waves in the water.	☐	☐	☐	☐	☐

Section B: Spontaneous Use of Imagery Scale (SUIS)

Please read each of the following descriptions and indicate the degree to which each is appropriate for you. Do not spend a lot of time thinking about each one, but respond based on your thoughts about how you do or do not perform each activity.

B1. If I catch a glance of a car that is partially hidden behind bushes, I automatically "complete it," seeing the entire car in my mind's eye.

- Never appropriate ☐
- Rarely appropriate ☐
- Appropriate about half of the time ☐
- Often appropriate ☐
- Always completely appropriate ☐

B2. **If I am looking for new furniture in a store, I always visualize what the furniture would look like in particular places in my home.**

- Never appropriate ☐
- Rarely appropriate ☐
- Appropriate about half of the time ☐
- Often appropriate ☐
- Always completely appropriate ☐

B3. **I prefer to read novels that lead me easily to visualize where the characters are and what they are doing instead of novels that are difficult to visualize.**

- Never appropriate ☐
- Rarely appropriate ☐
- Appropriate about half of the time ☐
- Often appropriate ☐
- Always completely appropriate ☐

B4. **Before I get dressed to go out, I first visualize what I will look like if I wear different combinations of clothes.**

- Never appropriate ☐
- Rarely appropriate ☐
- Appropriate about half of the time ☐
- Often appropriate ☐
- Always completely appropriate ☐

B5. **When going to a new place, I prefer directions that include detailed descriptions of landmarks (such as the size, shape and color of a gas station) in addition to their names.**

- Never appropriate ☐
- Rarely appropriate ☐
- Appropriate about half of the time ☐
- Often appropriate ☐
- Always completely appropriate ☐

B6. **When I think about visiting a relative, I almost always have a clear mental picture of him or her.**

- Never appropriate ☐
- Rarely appropriate ☐
- Appropriate about half of the time ☐
- Often appropriate ☐
- Always completely appropriate ☐

B7. **When relatively easy technical material is described clearly in a text, I find illustrations distracting because they interfere with my ability to visualize the material.**

- Never appropriate ☐
- Rarely appropriate ☐
- Appropriate about half of the time ☐
- Often appropriate ☐
- Always completely appropriate ☐

B8. **If someone were to tell me two-digit numbers to add (e.g., 24 and 31), I would visualize them in order to add them.**

- Never appropriate ☐
- Rarely appropriate ☐
- Appropriate about half of the time ☐
- Often appropriate ☐
- Always completely appropriate ☐

B9. **When I think about a series of errands I must do, I visualize the stores I will visit.**

- Never appropriate ☐
- Rarely appropriate ☐
- Appropriate about half of the time ☐
- Often appropriate ☐
- Always completely appropriate ☐

B10. **When I hear a radio announcer or DJ I've never actually seen, I usually find myself picturing what they might look like.**

- Never appropriate ☐
- Rarely appropriate ☐
- Appropriate about half of the time ☐
- Often appropriate ☐
- Always completely appropriate ☐

B11. **When I first hear a friend's voice, a visual image of him or her almost always springs to mind.**

- Never appropriate ☐
- Rarely appropriate ☐
- Appropriate about half of the time ☐
- Often appropriate ☐
- Always completely appropriate ☐

B12. **If I saw a car accident, I would visualize what had happened when later trying to recall the details.**

- Never appropriate ☐
- Rarely appropriate ☐
- Appropriate about half of the time ☐
- Often appropriate ☐
- Always completely appropriate ☐

Section C: About You

C1. **Please enter your participant ID:**

C2. **What is your age in years?**

C3. What is your first language?

A first language is the language a person is most familiar with and most accustomed to speaking.

Dutch ☐

English ☐

Other ☐

Other

C4. What was your level of understanding of the language during the experiment?

If you had difficulty understanding the instructions or audio stories, please explain why (language skills, concentration difficulties, clarity of instructions, etc.).

Very Poor: Minimal understanding, struggled to grasp even basic concepts. ☐

Poor: Limited understanding, struggled with comprehension of most content. ☐

Fair: Moderate understanding, managed to grasp some of the main points. ☐

Good: Solid understanding, comprehended the majority of the content with ease. ☐

Excellent: Perfect understanding, easily grasped all aspects of the language used in the experiment. ☐

C5. What is your gender?

Male ☐

Female ☐

Prefer not to say ☐

Other ☐

Other

LimeSurvey

C6. **How would you rate how well you see in your daily life? (with your glasses on or lenses in if you have corrected vision). Rate your vision using the following scale.**

- Excellent ☐
- Good ☐
- Fair ☐
- Poor ☐
- Very poor ☐

C7. **How would you rate how well you hear in your daily life? (with your hearing aid in if you have corrected hearing). Rate your hearing using the following scale.**

- Excellent ☐
- Good ☐
- Fair ☐
- Poor ☐
- Very poor ☐

C8. **Please note any feedback or comments you would like to add to your participation in the experiment and questionnaire.**

This could be how you felt during the experiment, a problem you encountered, or any important information you think the experimenter should take into account when analyzing your data.

Thank you for taking part!

LimeSurvey

Deze vragenlijst kan worden ingevuld in het Engels of het Nederlands.

Op geen enkel moment zullen we om uw identiteit vragen of uw IP-adres registreren. Verzamelde gegevens zullen worden versleuteld. Alle analyses zullen uitsluitend gebruikmaken van geanonimiseerde gegevens.

Help ons begrijpen hoe u uw 'innerlijke wereld' ervaart!

Sectie A: Vividness of Visual Imagery Questionnaire (VVIQ)

Voor elk item op deze vragenlijst probeer een visueel beeld te vormen en overweeg uw ervaring zorgvuldig. Voor elk beeld dat u ervaart, beoordeelt u hoe levendig het is met behulp van de vijfpuntsschaal. Als u geen visueel beeld heeft, beoordeel dan de levendigheid als 'Helemaal geen beeld, u weet alleen dat u aan het object denkt'. Gebruik alleen 'Volmaakt duidelijk en levendig als echt zien' voor beelden die echt zo levendig en helder zijn als echt zien. Let op: er zijn geen juiste of verkeerde antwoorden op de vragen, en het is niet noodzakelijk wenselijk om beelden te ervaren, of, als u dat wel doet, om levendigere beelden te hebben.

A1. Denk aan een familielid of vriend die u vaak ziet (maar die momenteel niet bij u is) en denk goed na over het beeld dat in u opkomt.

	Helemaal geen beeld, ik “weet” alleen dat ik aan het object denk	Vaag beeld	Matig realistisch en levendig	Realistisch en redelijk levendig	Volkomen realistisch, zo levendig als echt zien
De exacte contouren van gezicht, hoofd, schouders en lichaam.	☐	☐	☐	☐	☐
Karakteristieke hoofdhoudingen, lichaamshoudingen etc.	☐	☐	☐	☐	☐
De precieze houding, staplengte etc. bij het lopen.	☐	☐	☐	☐	☐
De verschillende kleuren die in bekende kleding worden gedragen.	☐	☐	☐	☐	☐

A2. Visualiseer een opkomende zon. Denk goed na over het beeld dat in u opkomt.

	Helemaal geen beeld, ik “weet” alleen dat ik aan het object denk	Vaag beeld	Matig realistisch en levendig	Realistisch en redelijk levendig	Volkomen realistisch, zo levendig als echt zien
De zon stijgt boven de horizon in een wazige lucht.	☐	☐	☐	☐	☐
De lucht klaart op en omringt de zon met blauwheid.	☐	☐	☐	☐	☐
Wolken. Een storm komt op met bliksemflitsen.	☐	☐	☐	☐	☐
Er verschijnt een regenboog.	☐	☐	☐	☐	☐

A3. Denk aan de voorkant van een winkel waar u vaak komt. Denk aan het beeld dat in u opkomt.

	Helemaal geen beeld, ik "weet" alleen dat ik aan het object denk	Vaag beeld	Matig realistisch en levendig	Realistisch en redelijk levendig	Volkomen realistisch, zo levendig als echt zien
De algehele uitstraling van de winkel vanaf de overkant van de weg.	☐	☐	☐	☐	☐
Een etalage met kleuren, vormen en details van individuele artikelen die te koop zijn.	☐	☐	☐	☐	☐
Je bent vlakbij de ingang. De kleur, vorm en details van de deur.	☐	☐	☐	☐	☐
Je komt de winkel binnen en gaat naar de balie. De balie assistent bedient je. Geld verandert van eigenaar.	☐	☐	☐	☐	☐

A4. Denk ten slotte aan een landelijke scène met bomen, bergen en een meer. Denk aan het beeld dat in u opkomt.

	Helemaal geen beeld, ik "weet" alleen dat ik aan het object denk	Vaag beeld	Matig realistisch en levendig	Realistisch en redelijk levendig	Volkomen realistisch, zo levendig als echt zien
De contouren van het landschap.	☐	☐	☐	☐	☐
De kleur en vorm van de bomen.	☐	☐	☐	☐	☐
De kleur en vorm van het meer.	☐	☐	☐	☐	☐
Een sterke wind waait op de bomen en op het meer waardoor er weerspiegelingen in het water ontstaan.	☐	☐	☐	☐	☐

Sectie B: Spontaneous Use of Imagery Scale (SUIS)

Lees de volgende beschrijvingen en duid aan in welke mate elke beschrijving op jou van toepassing is. Denk niet te lang na over elke beschrijving, maar antwoord op basis van jouw gedachten over hoe je de activiteit wel of niet zou uitvoeren.

B1. Wanneer ik een blik opvang van een auto die deels verborgen is achter struiken, dan "vervolledig" ik de auto automatisch door de auto in zijn geheel visueel voor te stellen in mijn hoofd.

- Nooit passend ☐
- Zelden passend ☐
- Ongeveer de helft van de tijd passend ☐
- Vaak passend ☐
- Altijd volledig passend ☐

B2. Wanneer ik in een winkel op zoek ben naar nieuwe meubels, maak ik mij altijd een voorstelling van hoe de meubels er zouden uitzien op bepaalde plaatsen in mijn huis.

- Nooit passend ☐
- Zelden passend ☐
- Ongeveer de helft van de tijd passend ☐
- Vaak passend ☐
- Altijd volledig passend ☐

B3. Ik verkies om romans te lezen die me er gemakkelijk toe brengen om voor te stellen waar de personages zijn en wat ze aan het doen zijn, in plaats van romans die moeilijk visueel voor te stellen zijn.

- ☐ Nooit passend
- ☐ Zelden passend
- ☐ Ongeveer de helft van de tijd passend
- ☐ Vaak passend
- ☐ Altijd volledig passend

B4. Voor ik mij aankleed om uit te gaan, stel ik mij eerst voor hoe ik er zal uitzien als ik de verschillende klerencombinaties draag.

- ☐ Nooit passend
- ☐ Zelden passend
- ☐ Ongeveer de helft van de tijd passend
- ☐ Vaak passend
- ☐ Altijd volledig passend

B5. Wanneer ik naar een nieuwe plaats ga, heb ik het liefst aanwijzingen die gedetailleerde beschrijvingen bevatten van oriëntatiepunten (zoals de grootte, vorm en kleur van een tankstation) naast de namen van die oriëntatiepunten.

- ☐ Nooit passend
- ☐ Zelden passend
- ☐ Ongeveer de helft van de tijd passend
- ☐ Vaak passend
- ☐ Altijd volledig passend

B6. Wanneer ik er aan denk een familielid te bezoeken, heb ik bijna altijd een duidelijk mentaal beeld van hem of haar.

- ☐ Nooit passend
- ☐ Zelden passend
- ☐ Ongeveer de helft van de tijd passend
- ☐ Vaak passend
- ☐ Altijd volledig passend

B7. **Wanneer relatief gemakkelijk technisch materiaal duidelijk beschreven wordt in een tekst, vind ik illustraties afleidend omdat ze interfereren met mijn bekwaamheid om het materiaal visueel voor te stellen.**

- Nooit passend ☐
- Zelden passend ☐
- Ongeveer de helft van de tijd passend ☐
- Vaak passend ☐
- Altijd volledig passend ☐

B8. **Als iemand me zou vragen om getallen die uit twee cijfers bestaan op te tellen (bv. 24 en 31), dan zou ik ze visueel voorstellen, wat me helpt om de getallen daarna op te tellen.**

- Nooit passend ☐
- Zelden passend ☐
- Ongeveer de helft van de tijd passend ☐
- Vaak passend ☐
- Altijd volledig passend ☐

B9. **Wanneer ik denk over een reeks boodschappen die ik moet doen, stel ik mij de winkels die ik ga bezoeken voor.**

- Nooit passend ☐
- Zelden passend ☐
- Ongeveer de helft van de tijd passend ☐
- Vaak passend ☐
- Altijd volledig passend ☐

B10. **Wanneer ik een radio-omroeper of een DJ hoor die ik nog nooit in het echt heb gezien, dan stel ik mezelf gewoonlijk voor hoe die er zou uitzien.**

- Nooit passend ☐
- Zelden passend ☐
- Ongeveer de helft van de tijd passend ☐
- Vaak passend ☐
- Altijd volledig passend ☐

LimeSurvey

B11. **Wanneer ik eerst de stem van een vriend of vriendin hoor, komt er bijna altijd een visueel beeld van hem of haar in mijn hoofd op.**

- Nooit passend ☐
- Zelden passend ☐
- Ongeveer de helft van de tijd passend ☐
- Vaak passend ☐
- Altijd volledig passend ☐

B12. **Wanneer ik een auto-ongeluk zou zien, zou ik mij een voorstelling maken van wat er gebeurd is wanneer ik later de details probeer te herinneren.**

- Nooit passend ☐
- Zelden passend ☐
- Ongeveer de helft van de tijd passend ☐
- Vaak passend ☐
- Altijd volledig passend ☐

Sectie C:

C1. **Voer alstublieft uw deelnemers-ID in:**

C2. **Wat is uw leeftijd in jaren?**

C3. **Wat is uw moedertaal?**

Een eerste taal is de taal waar een persoon het meest vertrouwd mee is en het meest aan gewend is om te spreken.

- Nederlands ☐
- Engels ☐
- Overige ☐

Overige

LimeSurvey

C4. Wat was uw niveau van begrip van de taal tijdens het experiment?

Als u moeite had met het begrijpen van de instructies of de audioverhalen, leg dan alstublieft uit waarom (taalvaardigheden, concentratieproblemen, duidelijkheid van de instructies, enzovoort).

- Zeer Slecht: Minimaal begrip, moeite om zelfs basisconcepten te begrijpen. ☐
- Slecht: Beperkt begrip, worstelde met het begrijpen van de meeste inhoud. ☐
- Redelijk: Matig begrip, slaagde erin om enkele van de belangrijkste punten te begrijpen. ☐
- Goed: Solide begrip, begreep het merendeel van de inhoud gemakkelijk. ☐
- Uitstekend: Perfect begrip, begreep alle aspecten van de gebruikte taal in het experiment moeiteloos. ☐

C5. Wat is uw geslacht?

- Mannelijk ☐
- Vrouwelijk ☐
- Geef liever geen antwoord ☐
- Overige ☐

Overige

C6. Hoe zou u beoordelen hoe goed u ziet in uw dagelijks leven (met uw bril op of lenzen in als u gecorrigeerd zicht heeft)? Beoordeel uw zicht met behulp van de volgende schaal.

- Uitstekend ☐
- Goed ☐
- Redelijk ☐
- Slecht ☐
- Zeer slecht ☐

LimeSurvey

C7. **Hoe zou u beoordelen hoe goed u hoort in uw dagelijks leven (met uw gehoorapparaat in als u gecorrigeerd gehoor heeft)? Beoordeel uw gehoor met behulp van de volgende schaal.**

- Uitstekend ☐
- Goed ☐
- Redelijk ☐
- Slecht ☐
- Zeer slecht ☐

C8. **Gelieve alle feedback of opmerkingen die u wilt toevoegen aan uw deelname aan het experiment en de vragenlijst te vermelden.**

Dit kan zijn hoe u zich voelde tijdens het experiment, een probleem dat u tegenkwam, of alle belangrijke informatie die u denkt dat de onderzoeker in overweging moet nemen bij het analyseren van uw gegevens.

Dank u voor uw deelname!
